# Mangrove-Derived Halotolerant Endophytic Bacteria Confer Strain-Specific and Consortium-Mediated Salt Tolerance in Peanut (*Arachis hypogaea* L.)

**DOI:** 10.3390/plants15203218

**Published:** 2026-10-20

**Authors:** Nagarathnamma Yammanuri, Subramanyam Reddy Chinreddy, Preetham Naik KT, Vishnu Babu Vallepu, Chandra Sekhar Akila, Chandra Obul Reddy Puli

**Affiliations:** 1 Department of Botany, School of Life Sciences, Yogi Vemana University, Kadapa 516005, India; nagarathna751@gmail.com (N.Y.); plantspreetham123@gmail.com (P.N.K.); vishnubabu20007@gmail.com (V.B.V.); 2 Department of Botany, SKR & SKR Government College for Women (A), Kadapa 516001, India; 3 Department of Biology, Gus R. Douglass Institute, West Virginia State University, Institute, WV 25112, USA; subramanyam.chinreddy@wvstateu.edu; 4 Department of Biotechnology, School of Life Sciences, Yogi Vemana University, Kadapa 516005, India; chandrasekhar9@yahoo.com

**Keywords:** peanut, mangrove endophytes, salinity, bacterial consortium, antioxidant defense, GUS-mediated colonization

## Abstract

Soil salinity is a major abiotic stress that severely limits crop productivity worldwide. This study evaluated the potential of halotolerant endophytes isolated from mangrove plants to enhance salinity tolerance in the peanut (*Arachis hypogaea* L. cv. JL24). A two-step approach, including screening and validation methods, was applied. Initially, six mangrove endophytes, ACR52, EAR173, EAR191, EAR195, EAL318, and ACL463, were tested for their capacity to colonize the non-host peanut. Histochemical detection of *gusA*-tagged derivatives confirmed early tissue-associated colonization of peanut seedlings by all six isolates. Seed inoculation of six endophytes improved germination under severe salinity, with ACR52 maintaining 83% final germination at 200 mM NaCl, compared with 67% in the uninoculated controls. The six-strain consortium accelerated germination, giving the highest germination at every NaCl level on Days 1 and 4. Among the isolates, EAL318 (*Bacillus subtilis*), EAR195 (*Proteus mirabilis*), and ACR52 (*Klebsiella quasipneumoniae* subsp. *similipneumoniae*) exhibited strong PGP traits and were selected for further evaluation. In greenhouse experiments with 300 and 500 mM NaCl, inoculated plants showed significantly higher plant height (32–40% increase), shoot dry weight (28–35% increase), and chlorophyll content (20–30% increase) compared with uninoculated controls. Under severe greenhouse salinity, ACR52 showed the most consistent preservation of reproductive performance, whereas C3 produced the broadest antioxidant response, with elevated CAT and POD activities and reduced H_2_O_2_ and MDA accumulation. These findings identify mangrove-derived endophytes as potential candidates for future field evaluation under saline-soil conditions.

## 1. Introduction

Salinity is a major abiotic stress that restricts plant growth and development by inducing osmotic stress, ionic toxicity, nutrient imbalance, and oxidative damage [1,2,3]. These effects disrupt key physiological processes, resulting in stunted growth, reduced yields, and significant losses in global agricultural productivity [4,5]. Salinity affects an estimated 20–33% of the world’s arid and irrigated lands and may increase to nearly 50% by 2050 [6]. Currently, about 1.4 billion hectares (10.7% of global land area) worldwide and 6.73 million hectares (2.1%) in India are salt-affected, posing a significant and growing threat to global food security [7].

Peanut (*Arachis hypogaea* L.), a major oilseed legume, is classified as moderately salt-tolerant, but is highly sensitive to salinity during germination and early growth [8,9]. Salinity-induced osmotic stress and ion toxicity (150–200 mM NaCl) reduce germination, seedling vigor, photosynthesis, biomass accumulation, reproductive development, and ultimately yield, leading to significant productivity losses [10,11,12]. Given the rising demand for peanut and increasing soil salinization, improving salinity tolerance through sustainable approaches is essential [13,14].

Mangrove ecosystems are a key source of stress-adapted plant-associated bacteria. These ecosystems experience fluctuating salinity, periodic tidal inundation, hypoxia, nutrient limitation, elevated temperatures, and other forms of stress, including oxidative stress. The combination of these environmental factors is a very strong ecological filter, enriching the microorganisms living in this environment with physiological and metabolic traits that help this community survive in highly saline, variable environments [15,16,17]. As a result, the bacteria obtained from mangroves are likely to adapt to multiple stresses better than those selected solely for their ability to grow in saline media.

Beyond the established advantages of conventional halotolerant plant growth-promoting rhizobacteria (PGPR), typically isolated from bulk soil or the rhizosphere, mangrove-derived endophytic bacteria may offer distinct strategies to enhance plant growth and stress tolerance [4,15,16,17]. Conventional PGPR can effectively promote plant growth; however, their efficacy depends largely on their ability to survive, establish, and colonize root surfaces within the dynamic and complex soil microbiome [18]. Endophytic bacteria not only have the ability to move within plants via roots and other plant tissues and form internal associations, but also establish asymptomatic associations within plant tissues. This intimate association with plant tissues facilitates the direct delivery of bacterial metabolites, enzymes, and other bioactive compounds to host cells, while the bacteria may at the same time be shielded from changes in soil conditions such as salinity, pH, water content, and competition among microbes [19]. Because of this, stress-adapted endophytes are likely to influence phytohormone balance, reduce stress ethylene via ACC deaminase, enhance nutrient uptake, regulate ion balance, enhance osmolyte production, enhance antioxidant activity, and modulate stress signals more consistently [15,20,21,22].

Of major interest here is the finding that the positive effects of mangrove-derived halophytic endophytes are not only limited to the host plants. Mangrove propagule endophytes, for example, help establish and support the growth of barley and rice in saline conditions [17,23]. Endophytes isolated from the mangrove *Avicennia marina* have successfully colonized tomato roots, reduced Na^+^ uptake and oxidative damage, and even improved plant growth under salt stress [24]. *Bacillus megaterium* JR-12 and *B. pumilus* GN-5, two types of bacteria isolated from the rhizosphere soil of Sundarban mangroves, have been found to improve osmotic potential, height, and biomass, and activate antioxidant enzymes in pearl millet plants at 150 mM NaCl [22]. *Salinicola salarius* AOL2, an endophyte from *Avicennia officinalis*, also showed high salt tolerance (up to 10% NaCl) and the ability to produce Indole-3-Acetic acid (IAA), dissolve phosphate, and release exoenzymes [15]. More recent research shows that microbial consortia from mangrove plants can confer salt tolerance to rice, mainly by regulating osmolyte accumulation, ionic balance, rhizosphere microbiome functionality, and salt-responsive gene expression [16]. Beyond this, endophytic bacteria originating from mangroves have also contributed to growth, yield, and stress tolerance in rice and *Arabidopsis* via stress signaling and root system structural modifications [25].

Taken together, the results described suggest that mangrove plants can be sources of biotechnologically important microbes that tolerate extreme salt concentrations (halotolerance), can colonize plants, and have multiple growth-promoting functions for plants (multiplicity of functions). That is why *Excoecaria agallocha* (L.) and *Aegiceras corniculatum* (L.) were chosen as sources of endophytic bacteria, and peanut plants were used as non-host plants to evaluate whether these stress-adaptive microbes might be used to increase the salt tolerance of salt-sensitive peanut plants, which are also economically important. Mangrove plants were collected from the Duggirajapatnam region of Andhra Pradesh, India, where the soil pH ranged from 8.93 and the EC value was 12.2 mS.

While halotolerant PGPR have become popular for salinity management, the focus on mangrove-derived endophytes to improve peanut tolerance has been limited. Most researchers have relied on native crop or rhizosphere isolates, whereas transferring mangrove endophytes to non-host crops across different ecosystems is not well known. In general, the roles of bacterial groups in enhancing peanut nodulation, antioxidant protection, osmotic adjustment, and production under high salinity remain a challenge. Accordingly, this study aimed to isolate and characterize halotolerant endophytic bacteria from mangrove plants, *Excoecaria agallocha* and *Aegiceras corniculatum*, and to evaluate their plant growth-promoting traits and efficacy in enhancing salinity tolerance in a non-host crop, peanut (cv. JL-24).

## 2. Results

### 2.1. Sequential Screening and Selection of Halotolerant Endophytic Bacteria

**Figure 1:**
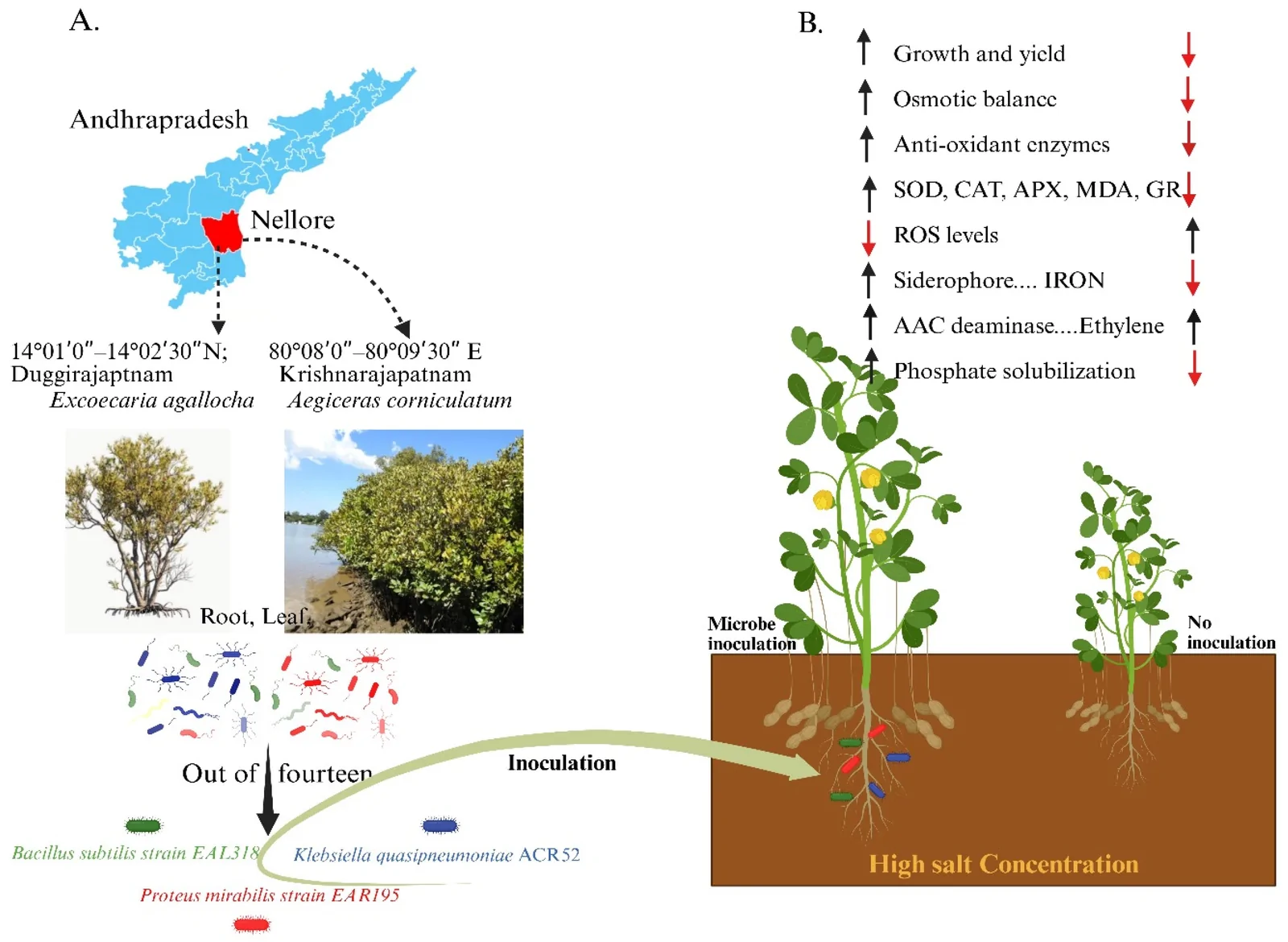
(**A**) Collection and isolation of HTPGPB from Mangrove ecosystem Nellore (Dt.) Andhra Pradesh, India. (**B**) Proposed hypothesis of inoculated bacteria that improved peanut saline stress.

A two-step approach, including screening and validation methods, was applied. First, among the halotolerant endophytic bacteria, six different isolates—ACR52 (PV657114), EAR173 (PV660264), EAR191(PV661994), EAR195(PV656438), EAL318 (PV656428), and ACL463—were evaluated individually for early peanut colonization and subsequently screened, individually and as C6, for effects on seed germination and seedling performance under salinity. These isolates exhibited notable traits, including enhanced phytohormone production, increased nutrient solubilization, and improved stress tolerance (unpublished). An uninoculated (surface-sterilized, uninoculated) peanut control and all the endophyte-inoculated and uninoculated seedlings received 0, 50, 100, 150, 200, and 250 mM NaCl treatments (Figure 1).

### 2.2. Histochemical Confirmation of Early Colonization of Peanut by Mangrove-Derived Endophytic Bacteria

Seed-imprint plates prepared from surface-sterilized peanut seeds and plating of the final rinse of the surface sterilization showed no visible microbial colony development after 48 h of incubation, confirming the effective removal of culturable surface-associated microorganisms under the sterilization conditions used. Before assessing their plant growth-promoting abilities, the capacity of the six selected candidate mangrove endophyte isolates to colonize a non-native host, peanut, was examined using GUS reporter-tagged derivatives, which were detected histochemically using X-Gluc and examined by bright-field microscopy. Peanut seedlings inoculated with ACR52, EAR173, EAR191, EAR195, EAL318, and ACL463 showed visible reporter activities in the roots and stems. At the same time, uninoculated controls showed no visible reporter activity upon microscopic examination of the plant tissues.

**Figure 2:**
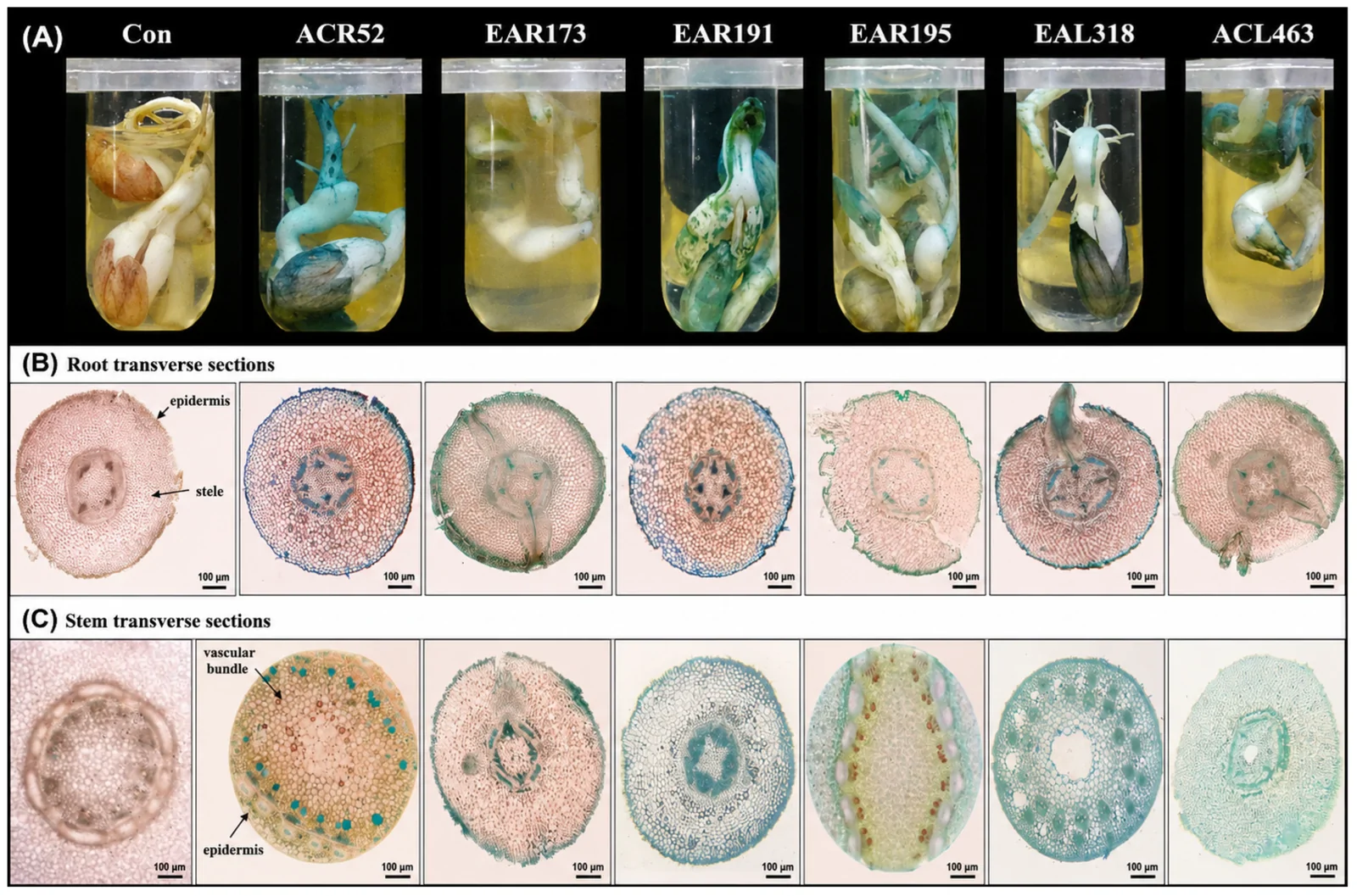
Histochemical confirmation of early colonization of peanut seedlings by *gusA*-tagged mangrove-derived endophytic bacteria. (**A**) Whole-seedling X-Gluc staining of peanut cv. JL24 seedlings inoculated with *gusA*-tagged derivatives of ACR52, EAR173, EAR191, EAR195, EAL318, and ACL463, with uninoculated seedlings used as the control (Con). (**B**) Representative root transverse sections showing isolate-dependent reporter-associated staining in epidermal, cortical, and stele-associated tissues. (**C**) Representative stem transverse sections showing reporter-associated staining in epidermal, cortical, and vascular tissues. Blue-green staining indicates the presence of the inoculated *gusA*-tagged bacteria. No comparable staining was observed in the uninoculated control. The images demonstrate that all six isolates were capable of establishing early tissue-associated colonization in peanut seedlings, although the spatial distribution of staining varied among isolates. Scale bars = 100 µm.

Histochemical X-Gluc staining confirmed early tissue-associated colonization of peanut cv. JL24 by all six *gusA*-tagged endophytic bacterial isolates (Figure 2). Whole seedlings inoculated with ACR52, EAR173, EAR191, EAR195, EAL318, and ACL463 showed blue-green reporter-associated staining, whereas no comparable staining was observed in the uninoculated control. Root and stem transverse sections revealed isolate-dependent localization in epidermal, cortical, vascular, and stele-associated tissues. ACR52, EAR173, and EAR191 showed both peripheral and internal localization, while EAR195 exhibited discontinuous vascular-associated staining. EAL318 and ACL463 were also detected in cortical and vascular regions. These observations demonstrate that all six isolates colonized peanut tissues at the assessed seedling stage, although the assay did not quantify colonization efficiency or long-term persistence.

### 2.3. Primary Seedling-Stage Screening of Individual Isolates and the Six-Strain Consortium

**Figure 3:**
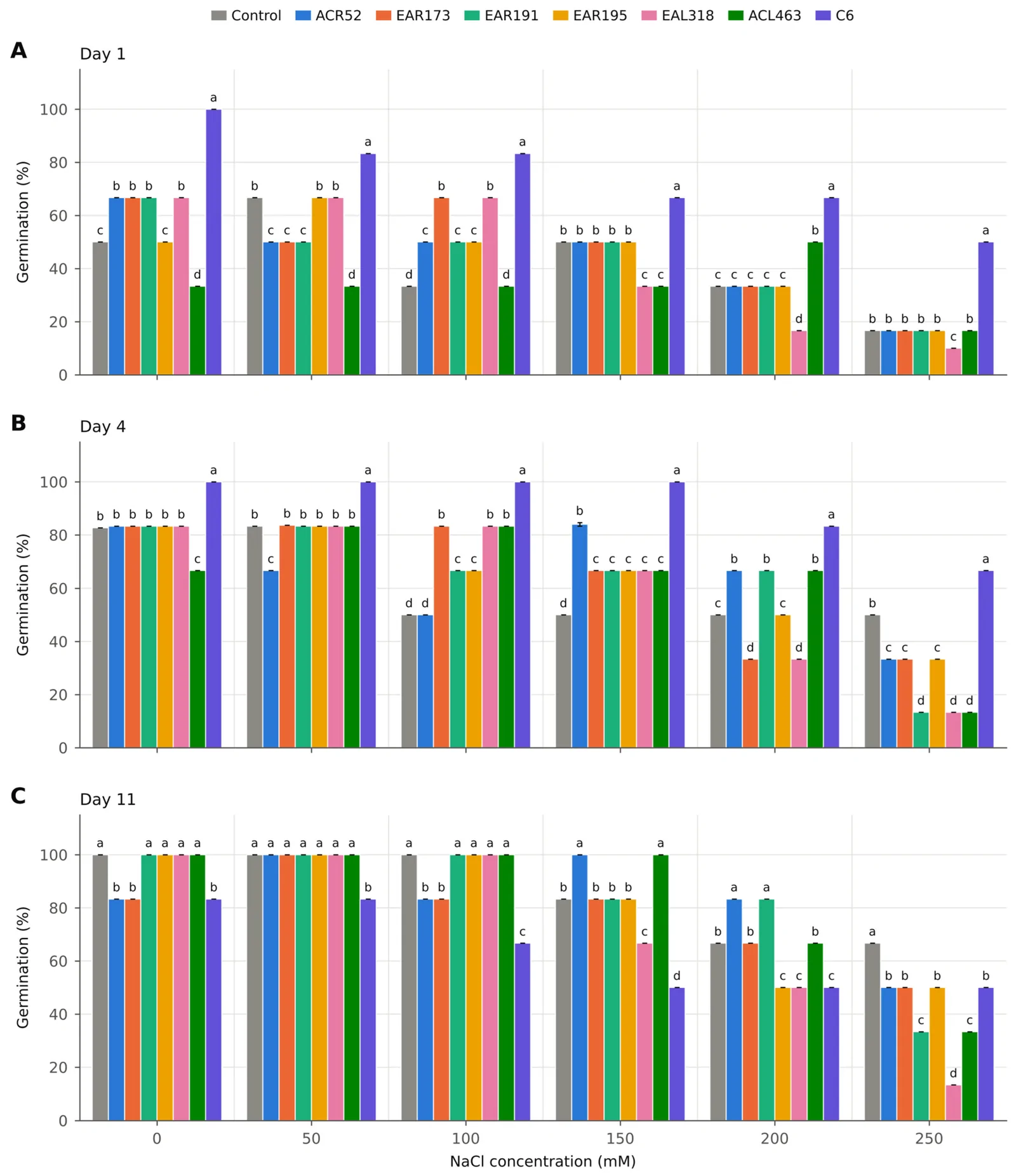
Effects of individual mangrove-derived endophytic bacterial isolates and the six-strain consortium C6 on peanut seed germination under NaCl stress. Germination percentages of peanut cv. JL24 were recorded on (**A**) Day 1, (**B**) Day 4, and (**C**) Day 11 under 0, 50, 100, 150, 200, and 250 mM NaCl. Treatments comprised the uninoculated control, ACR52, EAR173, EAR191, EAR195, EAL318, ACL463, and the six-strain consortium C6 containing all six isolates. Values represent mean ± SE of [*n* = 3] three independent Petri dishes per treatment combination, with each Petri dish considered one experimental unit. Data for each observation day were analyzed by two-way ANOVA using bacterial treatment and NaCl concentration as fixed factors, followed by Tukey’s multiple-comparison test. Different letters indicate significant differences among treatment combinations at *p* < 0.05.

The salinity negatively affected peanut germination, seedling vigor, growth, and biomass accumulation by progressively reducing each. Still, the degree of reduction varied with the applied bacterial treatment, NaCl concentration (0, 50, 100, 150, 200, and 250 mM), and the timing of assessment. In the first screening stage, single isolates (ACR52, EAR173, EAR191, EAR195, EAL318, and ACL463), the six-strain consortium C6, and an uninoculated control were evaluated. Salt concentration gradually inhibited peanut germination, but the endophyte inoculation somewhat counteracted this effect, especially under high-salinity conditions (Figure 3).

**Figure 4:**
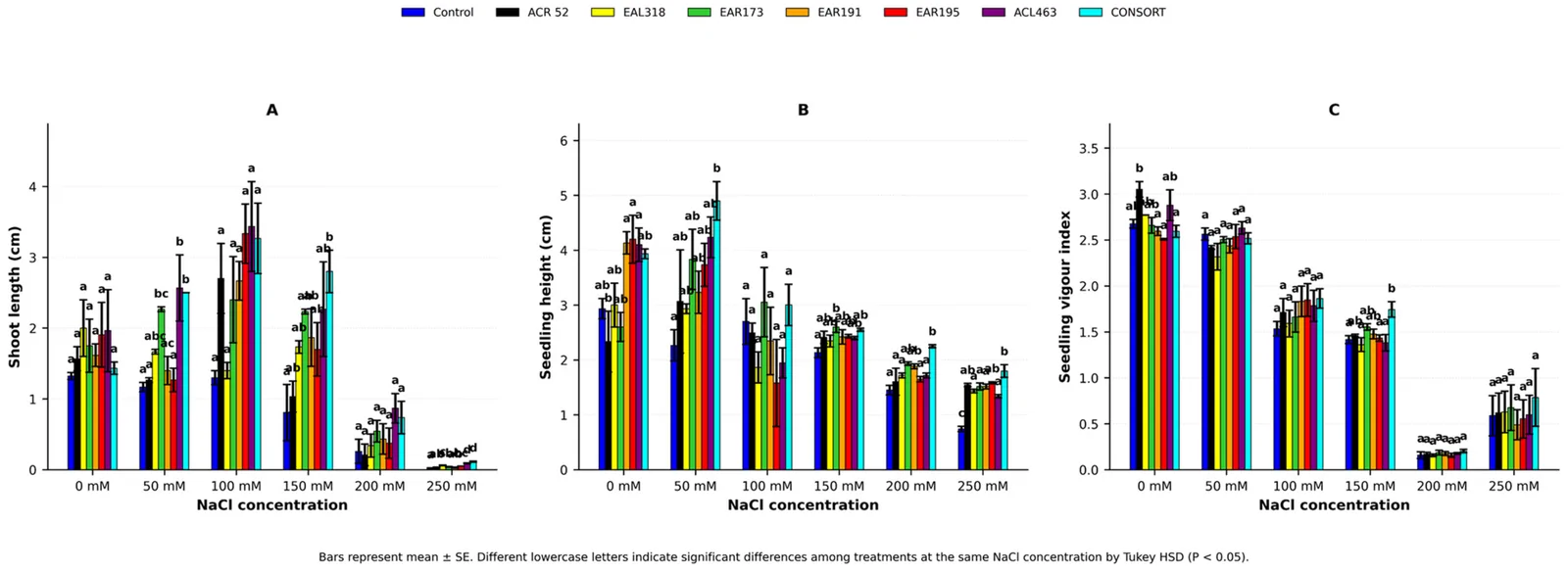
Effects of mangrove-derived endophytic bacterial inoculation on seedling vigor and seedling length of peanut under NaCl stress. Surface-sterilized seeds of peanut cv. JL24 were inoculated individually with ACR52, EAR173, EAR191, EAR195, EAL318, or ACL463, or with the six-strain consortium C6 comprising all six isolates; uninoculated seeds served as the control. (**A**) Seedling shoot length. (**B**) Seedling height. (**C**) Seedling vigor index was determined on Day 11th after exposure to 0, 50, 100, 150, 200, and 250 mM NaCl. Values represent mean ± SE of three independent Petri dishes per bacterial treatment × NaCl combination (n = 3), with each Petri dish considered an independent biological replicate and experimental unit. Measurements obtained from seedlings within the same Petri dish were averaged to generate a single dish-level observation. Data were analyzed by two-way ANOVA with bacterial inoculation and NaCl concentration as fixed factors, including their interaction, followed by Tukey’s multiple-comparison test. Different lowercase letters indicate significant differences at *p* < 0.05.

By the 11th day, ACR52-inoculated seeds maintained 83% germination at 200 mM NaCl, compared with 66% in the uninoculated control (Figure 3C). At 250 mM NaCl, ACR52-treated seeds still had 50% germination, which was lower than the control (66.7%). Based on these results, ACR52 appears reliable for seedling development up to 200 mM NaCl. In contrast, C6 accelerated germination, giving the highest germination at every NaCl level on Days 1 and 4 (Figure 3A,B), although its final germination on Day 11 did not exceed that of the control. C6 also improved seedling vigor, growth, and biomass traits (Figure 4, Figures S1 and S2).

The individual isolates exhibited complementary responses. ACR52 was mostly associated with germination protection, EAL318 was best at conserving root growth and root dry matter, and EAR195 was best at preserving growth and biomass-related traits. It performed well across the screening parameters. Certain isolates have different effects. The overall effectiveness of each isolate at different germination stages, as well as its effects on growth and biomass traits, was evaluated. Because of this, ACR52, EAL318, and EAR195 were considered for further work whereas the isolates EAR173, EAR191, and ACL463 were dropped from the list, as their performance across different germination stages, as well as in vigor, growth, and biomass, was less consistent than the elected isolates.

In the next greenhouse trial, the chosen bacteria were paired again, forming a novel three-bacteria consortium called C3. That is why the C6 treatment tested at the seedling stage and the C3 treatment tested during the vegetative and reproductive stages differed in composition.

### 2.4. Endophytic Treatment Confers Distinct Root, Shoot, and Photosynthetic Advantages Under Moderate and Severe Salinity

In the second stage, three bacterial isolates—ACR52, EAL318, and EAR195—were identified and carried forward for validation based on their cumulative performance in seedling emergence, vigor, and plant growth. These isolates were tested individually and as a consortium (C3, a mix of three isolates).

**Figure 5:**
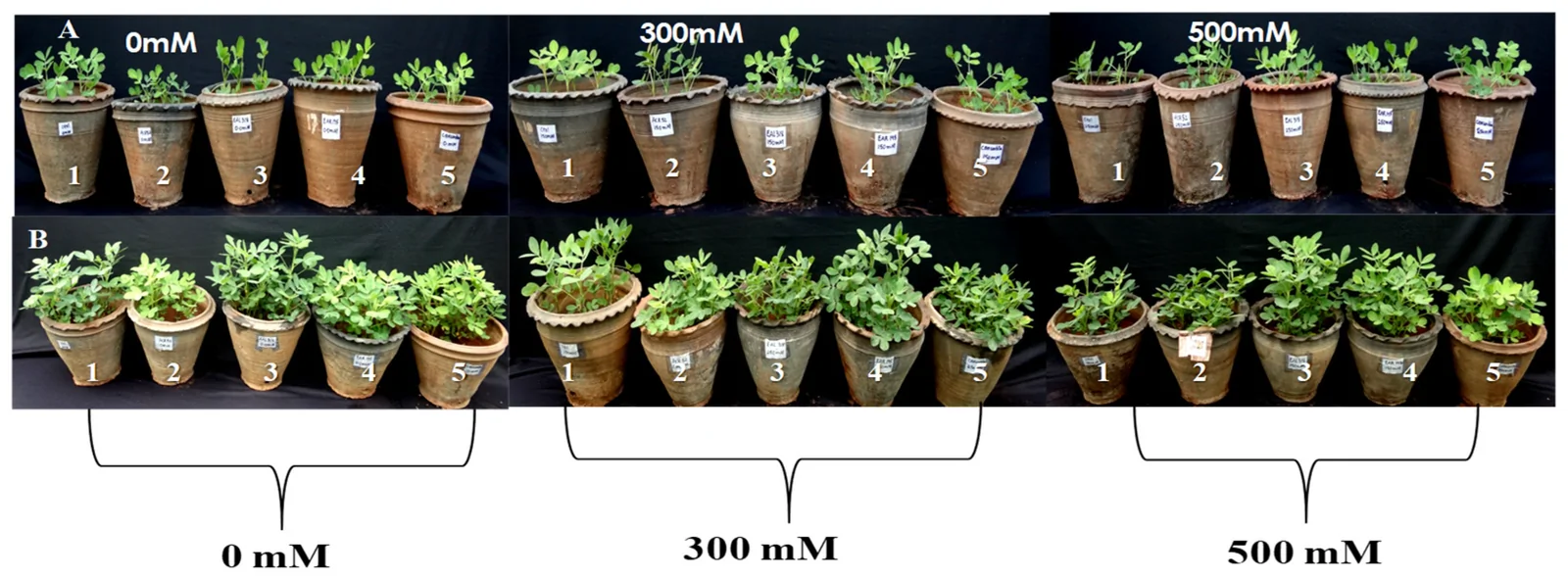
Representative photographs showing the effects of salinity and bacterial inoculation on peanut growth during the vegetative stage. Peanut cv. JL24 plants were grown under 0, 300, and 500 mM NaCl and photographed at (**A**) 40 days after sowing (DAS) and (**B**) 60 DAS. Within each salinity level, the treatments were: 1, uninoculated control; 2, ACR52; 3, EAR195; 4, EAL318; and 5, the three-strain consortium C3 comprising ACR52, EAR195, and EAL318. The images illustrate the treatment-associated differences in plant vigor, shoot development, and overall growth under increasing salinity stress.

The inoculated peanut plants were tested at the vegetative and reproductive phases at 0, 300, and 500 mM NaCl concentrations (Figure 5).

**Figure 6:**
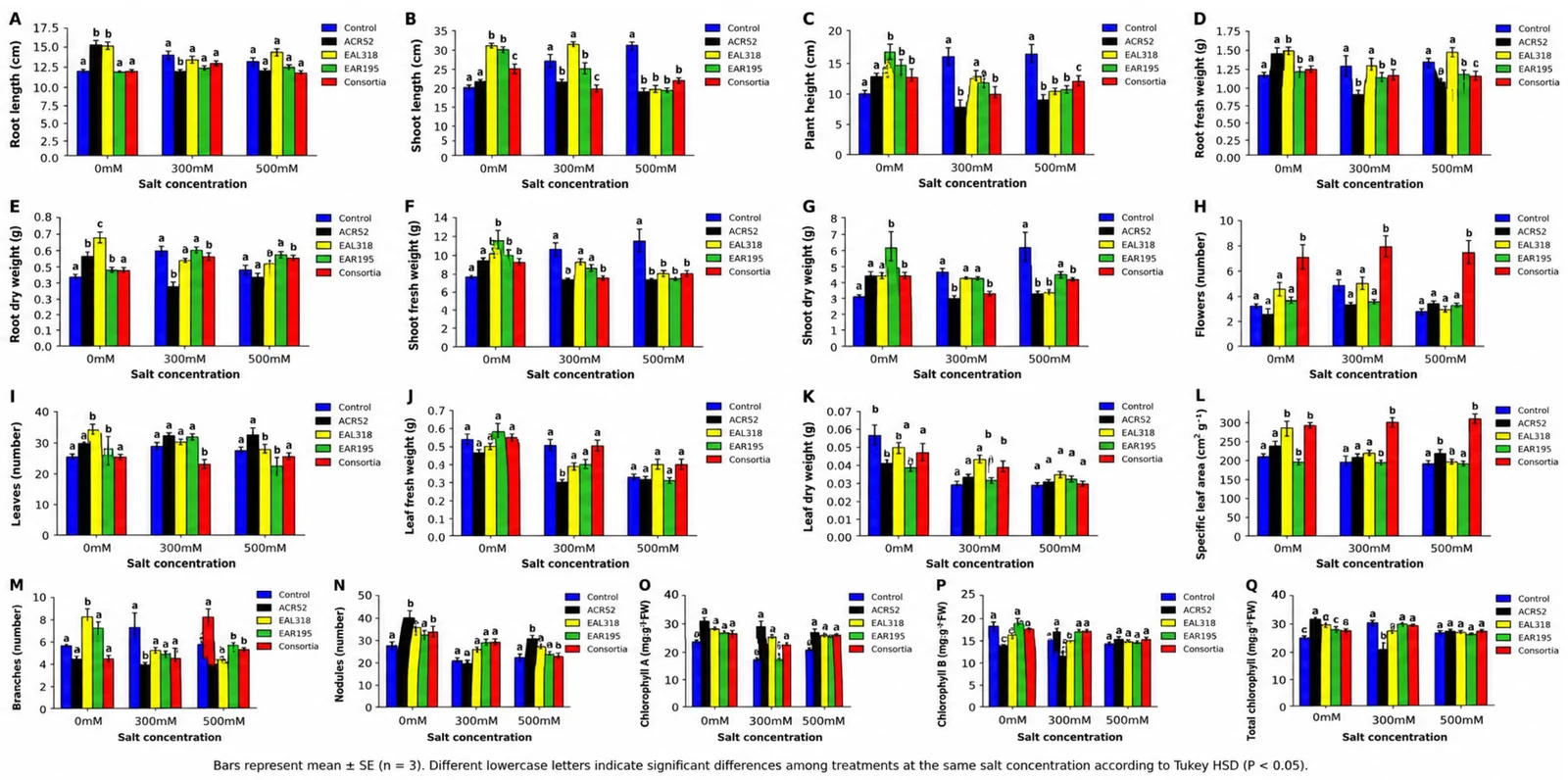
Effects of selected mangrove-derived endophytic bacteria and the three-strain consortium C3 on the growth, biomass, leaf, and reproductive traits of peanut under salinity. Peanut cv. JL24 plants were inoculated with ACR52, EAL318, EAR195, or the three-strain consortium C3 comprising ACR52, EAL318, and EAR195; uninoculated plants served as the control. Plants were evaluated under 0, 300, and 500 mM NaCl. The measured parameters were: (**A**) root length, (**B**) shoot length, (**C**) plant height, (**D**) root fresh weight, (**E**) root dry weight, (**F**) shoot fresh weight, (**G**) shoot dry weight, (**H**) flower number, (**I**) number of leaves, (**J**) leaf fresh weight, (**K**) leaf dry weight, (**L**) specific leaf area, (**M**) branches, (**N**) number of root nodules, (**O**) Chlorophyll A, (**P**) Chlorophyll B, and (**Q**) total chlorophyll content. Values represent mean ± SE of three independent pots per bacterial treatment × NaCl combination (n = 3), with each pot considered the experimental unit. Where more than one plant was assessed within a pot, the measurements were averaged to obtain a single pot-level observation. Data were analyzed by two-way ANOVA with bacterial inoculation and NaCl concentration as fixed factors, including their interaction, followed by Tukey’s multiple-comparison test. Different lowercase letters above the bars indicate significant differences among treatment combinations at *p* < 0.05.

**Figure 7:**
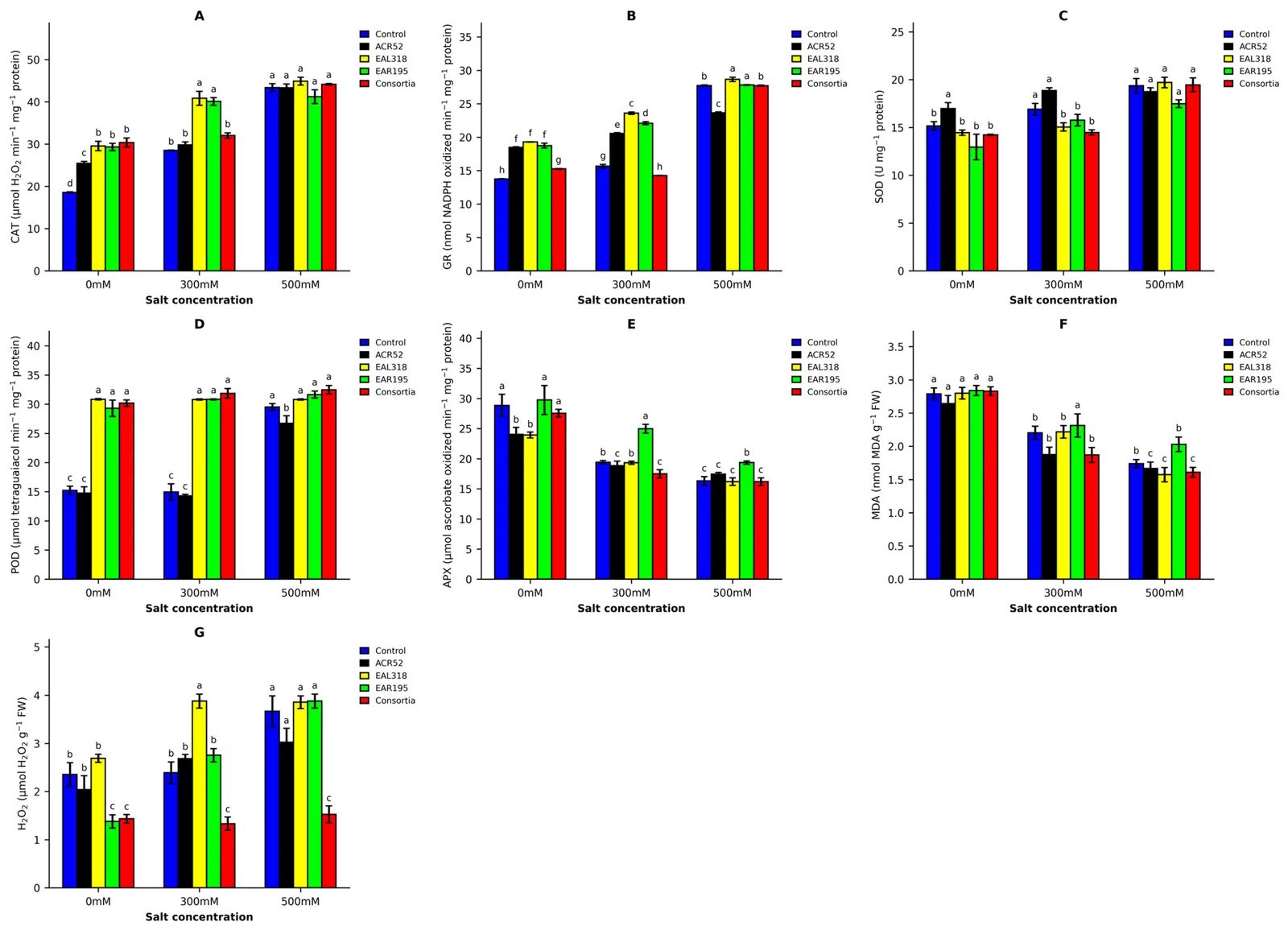
Effects of selected mangrove-derived endophytic bacteria and C3 on leaf antioxidant-enzyme activities and oxidative-damage indicators of peanut under salinity. The measured parameters were: (**A**) catalase (CAT), (**B**) glutathione reductase (GR) activity, (**C**) superoxide dismutase (SOD) activity, (**D**) peroxidase (POD), (**E**) ascorbate peroxidase (APX) activity, (**F**) malondialdehyde (MDA) content, and (**G**) H_2_O_2_ content. Values represent mean ± SE of three independent biological replicates per treatment combination (n = 3), with each replicate consisting of leaf tissue collected from three plants from each experimental pot. Each assay was performed in technical triplicate, and the technical readings were averaged before statistical analysis. Data were analyzed using two-way ANOVA followed by Tukey’s multiple-comparison test. Different letters indicate significant differences at *p* < 0.05.

Higher salinity altered plant root development, shoot growth, biomass accumulation, and photosynthetic productivity (Figure 6 and Figure 7), but different microbial applications improved some features better than others. In the absence of salt, EAL318 was the most obvious microbe in promoting root growth, whilst the consortium and individual strains showed higher values in various plant and leaf development and shoot components (Figure 7). At a NaCl concentration of 300 mM, ACR52 inoculated peanut plants maintained the highest chlorophyll a level, while EAR195 was most responsive in leaf number and shoot-associated growth. Overall, these results illustrate that moderate salinity led to the separation of the treatment groups based on their main physiological effects rather than the emergence of a single, universally optimal treatment.

At 500 mM NaCl, EAL318 and the endophyte consortium were more effective at keeping root and shoot lengths intact, whereas EAR195 and ACR52 showed comparatively higher total biomass (Figure 6). EAR195 also maintained a much higher level of chlorophyll a than the uninoculated control under extreme salinity, but the consortium and ACR52 had broader effects on leaf water content and photosynthetic pigments across the various salinity treatments. Therefore, first and foremost, EAL318 was linked to protecting the root system from damage and EAR195 to maintaining the shoot and the plant’s total biomass, while ACR52 was the main one for safeguarding the photosynthetic machinery.

In addition to mitigating salinity effects to some extent, microbe inoculation also reduced salinity’s effects on nodulation and flowering (Figure 7). Compared with other treatments during the reproductive phase, the consortium exhibited a more complete, integrated response, combining high numbers of nodules and flowers. Still, at very high salinity levels, ACR52 maintained a relatively high level of nodulation. Overall, the consortium was the best at protecting the most diverse traits, whereas individual bacterial isolates showed rather specific features only at the salinity level that affected them most strongly.

### 2.5. Strain-Specific Antioxidant and Osmotic-Adjustment Strategies Reduce Salt-Induced Oxidative Damage

Bacterial treatments induced distinct antioxidant responses in plants subjected to severe salinity (Figure 7). The highest POD activity of 32.48 ± 0.73 (µmol tetraguaiacol min^−1^ mg^−1^ protein) was observed in the consortia C3-treated plants, while the lowest H_2_O_2_ accumulation of 1.53 ± 0.18 (1.53 ± 0.18 µmol H_2_O_2_ g^−1^ FW) was observed in this treatment at 500 mM NaCl. Also, the MDA content was comparatively low in C3-treated plants (1.61 ± 0.07 nmol MDA g^−1^ FW), indicating less cellular oxidative injury. On the contrary, the highest level of APX activity was observed in EAR195 (19.36 ± 0.26 µmol ascorbate oxidized min^−1^ mg^−1^ protein). At the same time, EAL318 and EAR195 had a higher level of GR activity (28.65 ± 0.32 and 27.81 ± 0.05 nmol NADPH oxidized min^−1^ mg^−1^ protein, respectively). The CAT activity remained high in several treatment groups at 500 mM NaCl, with EAL318 and C3 being the highest at 44.88 ± 0.93 and 44.14 ± 0.19 µmol H_2_O_2_ min^−1^ mg^−1^ protein, respectively.

The three different endophytes used in this study triggered distinct protective mechanisms in peanuts to cope with salinity stress. While all peanut plants responded to salt stress by producing antioxidants and osmoprotectants (Figure S3), it was clearly the endophytes that were responsible for differences in the types of compounds produced by the plants. When the peanut plants were subjected to 500 mM NaCl, the endophyte consortium-inoculated peanuts showed the highest peroxidase and catalase activities while exhibiting the lowest lipid peroxidation and hydrogen peroxide levels.

Compared with other endophytes, the bacterial strain EAL318 inoculated into peanuts showed very strong superoxide dismutase activity and, together with another isolate EAR 195, showed very high glutathione reductase activity. Under very strong salinity, EAR195 had the highest APX enzyme activity. Based on this, it appears that EAL318 primarily protects plants against superoxide and maintains glutathione levels, whereas EAR195 eliminates peroxy radicals via APX and GR. The different characteristics and activities of individual and consortium endophytes indicate that they provide a range of total antioxidant capacities for peanut, whereas consortium-inoculated plants produce all of the essential components of the antioxidant system that enable the detoxification of stress-induced free radicals.

The treatments differed much in their effects on osmotic adjustments and carbon metabolism (Figure S3). At 500 mM NaCl, C3 maintained free proline at 38.05 ± 0.05 µmol g^−1^ FW and soluble sugars at 10.77 ± 0.69 µmol g^−1^ FW, whereas EAR195 maintained 21.07 ± 1.52 mg g^−1^ FW for free amino acids. Significant treatment differences are indicated by Tukey grouping in Figure S2. ACR52 seemed to retain a somewhat higher level of free amino acids. Overall, the results suggest that the consortium is mainly involved in both antioxidant and osmotic adjustment defenses, whereas for the other strains, EAL318′s role was mostly the enhancement of the enzymatic system responsible for the scavenging of ROS; EAR195 is characterized by its high level of antioxidant activities and a high potential for a carbon metabolism change; ACR52 contributes to the peanut’s ability to maintain a state of physiological and metabolic balance under salt stress.

Root antioxidant responses further demonstrated treatment-specific regulation of oxidative stress (Supplementary Table S1). At 500 mM NaCl, EAL318 exhibited the highest root SOD activity (26.86 ± 0.71 μmol g^−1^ FW), whereas EAR195 maintained the highest APX activity (19.68 ± 0.78 μmol g^−1^ FW). In contrast, C3 produced the highest CAT (46.75 ± 0.65 μmol g^−1^ FW) and POD (38.84 ± 0.48 μmol g^−1^ FW) activities and simultaneously maintained the lowest H_2_O_2_ (1.59 ± 0.13 μmol g^−1^ FW) and MDA (1.37 ± 0.06 μmol g^−1^ FW) contents. These root-level responses indicate that the bacterial treatments differentially affected individual antioxidant components, whereas C3 was associated with a broader peroxide-detoxification response accompanied by reduced oxidative-damage indicators.

### 2.6. Individual Strains and the Consortium Differ in Yield Promotion and Yield Retention Under Salinity

**Figure 8:**
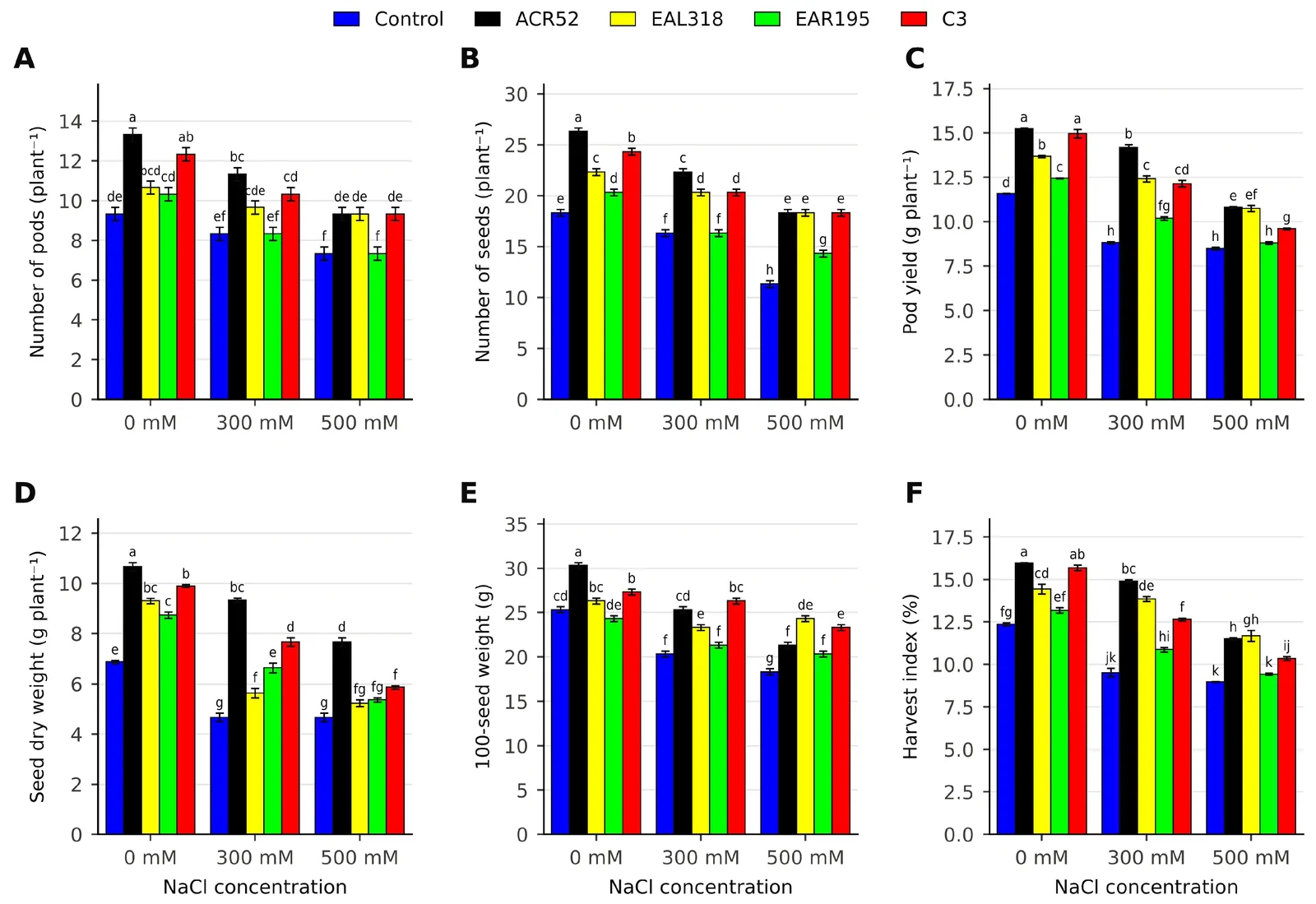
Effects of selected mangrove-derived endophytic bacterial isolates and the three-strain consortium C3 on reproductive and yield traits of peanut under salinity. Peanut cv. JL24 plants were inoculated individually with ACR52, EAL318, or EAR195, or with the three-strain consortium C3 comprising ACR52, EAL318, and EAR195; uninoculated plants served as the control. Plants were evaluated under 0, 300, and 500 mM NaCl. The measured traits were: (**A**) number of pods per plant, (**B**) number of seeds per plant, (**C**) pod yield per plant (g plant^−1^), (**D**) seed dry weight (g plant^−1^), (**E**) 100-seed weight (g), and (**F**) harvest index (%). Values represent mean ± SE of three independent pots per bacterial treatment × NaCl combination (n = 3), with each pot considered an independent biological replicate and experimental unit. When more than one plant was assessed within a pot, the measurements were averaged to obtain a single pot-level observation. Data were analyzed by two-way ANOVA with bacterial inoculation and NaCl concentration as fixed factors, including their interaction, followed by Tukey’s HSD test at *p* < 0.05. Different lowercase letters above the bars indicate significant differences among treatment combinations (Tukey’s HSD, *p* < 0.05).

Salinity progressively reduced reproductive performance and yield, with the magnitude of the reduction varying strongly across bacterial treatments (Figure 8 and Figure S4). ACR52 showed the most consistent preservation of reproductive performance under severe salinity. At 500 mM NaCl, ACR52-treated plants produced 9.33 ± 0.33 pods plant^−1^ compared with 7.33 ± 0.33 in the uninoculated control and maintained 18.33 ± 0.33 seeds plant^−1^ compared with 11.33 ± 0.33 in the control. Pod yield was correspondingly higher in ACR52-treated plants (10.82 ± 0.04 g plant^−1^) than in the control (8.50 ± 0.07 g plant^−1^), while seed dry weight was maintained at 7.67 ± 0.17 g plant^−1^ compared with 4.67 ± 0.17 g plant^−1^ in uninoculated plants. Harvest index also remained higher in ACR52-treated plants (11.51 ± 0.05%) than in the control (8.98 ± 0.02%). Tukey grouping confirmed the separation of these treatment combinations at *p* < 0.05.

### 2.7. Multivariate Integration of Growth, Stress-Defense and Yield Responses

**Figure 9:**
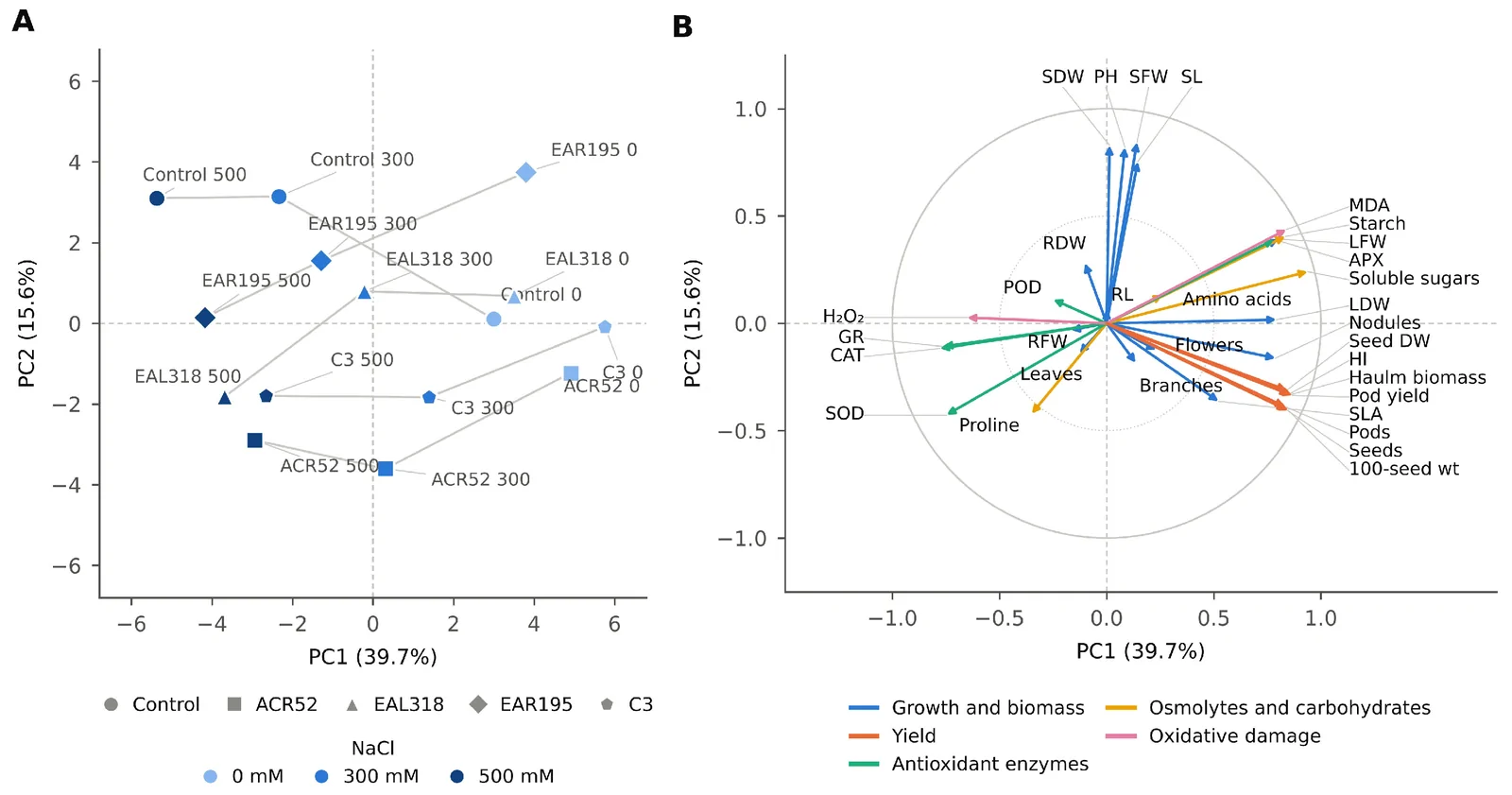
Principal component analysis (PCA) of morphological, physiological, biochemical, and reproductive traits of peanut cv. JL24 under salinity and bacterial inoculation. (**A**) Score plot of the 15 treatment combinations (uninoculated control, ACR52, EAL318, EAR195, and the three-strain consortium C3, each at 0, 300, and 500 mM NaCl). Marker shape indicates the bacterial treatment and shading the NaCl level; gray lines connect the three salinity levels of each treatment. (**B**) Loading plot of 32 variables, colored by trait group. Arrow direction and length show each variable’s correlation with PC1 and PC2; the outer circle marks *r* = 1 and the dotted circle *r* = 0.5. PCA was performed on standardized treatment-combination means (*n* = 3). RL, root length; SL, shoot length; PH, plant height; RFW/RDW, root fresh/dry weight; SFW/SDW, shoot fresh/dry weight; LFW/LDW, leaf fresh/dry weight; SLA, specific leaf area; HI, harvest index; Seed DW, seed dry weight; CAT, catalase; GR, glutathione reductase; SOD, superoxide dismutase; POD, peroxidase; APX, ascorbate peroxidase; MDA, malondialdehyde.

The first principal component (PC1) differentiated productive from salt-injured treatments, whereas the second principal component (PC2) explained the variation in biochemical strategies for the salts (Figure 9). In short, plant traits like growth, chlorophyll biomass, and yield clustered together, while MDA and H_2_O_2_ were positioned in opposite directions. This biplot arrangement of antioxidant enzymes and osmolytes suggests that higher defense activity is not necessarily equated with oxidative damage. Finally, from a practical point of view, the biplot positions of ACR52, EAL318, EAR195, and the consortium should be used directly to name their features.

**Figure 10:**
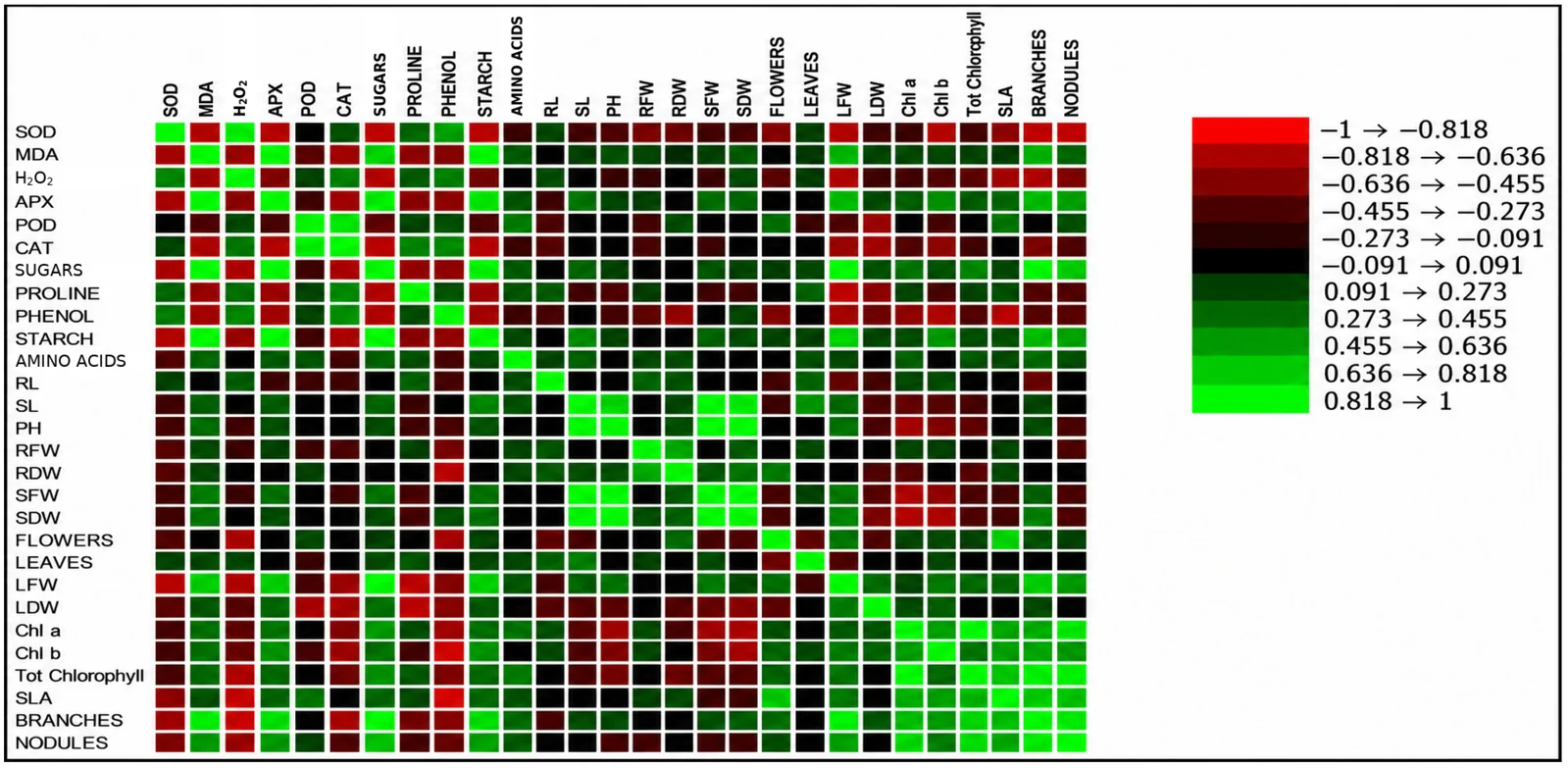
Pearson correlation matrix showing associations among morphological, physiological, biochemical, and reproductive traits of peanut under salinity and bacterial inoculation. Correlation coefficients were calculated among growth, biomass, leaf, photosynthetic pigments, antioxidant enzymes, osmolytes, oxidative damage, nodulation, and reproductive traits.

Yield components were strongly positively correlated with biomass and photosynthetic traits, suggesting that treatments that maintained the biomass and chlorophyll were also those that best maintained reproductive functions (Figure 10). In contrast, H_2_O_2_ content was negatively correlated with most yield traits (r = −0.51 to −0.56), indicating that peroxide accumulation was associated with productivity loss. MDA content, however, declined with increasing salinity and was therefore positively associated with yield traits, suggesting that MDA was not a reliable indicator of salt-induced damage under the present conditions. Antioxidant enzymes and osmolytes were not consistently correlated because their levels reflect an induced defense mechanism whose strength depends on salinity and bacterial treatment. These correlations support, but do not establish, causal relationships among antioxidant activation, damage reduction, and yield maintenance.

Peanut cv. JL24 plants were inoculated with ACR52, EAL318, EAR195, or the three-strain consortium C3 comprising ACR52, EAL318, and EAR195; uninoculated plants served as the control. Treatments were evaluated under 0, 300, and 500 mM NaCl. The biplot shows the distribution of treatment combinations and the loadings of growth, biomass, photosynthetic pigment, antioxidant enzyme, osmolyte, oxidative damage, and yield-related variables. The first and second principal components explained 39.7% and 15.6% of the total variance, respectively. Arrow direction indicates the relationship of each variable with the principal components, whereas arrow length reflects its relative contribution to the ordination.

Peanut cv. JL24 plants were inoculated with ACR52, EAL318, EAR195, or the three-strain consortium C3 (ACR52, EAL318, and EAR195); uninoculated plants served as the control. Treatments were evaluated under 0, 300, and 500 mM NaCl. The color scale represents the direction and magnitude of Pearson’s correlation coefficient (r), ranging from −1 to +1. Positive correlations indicate that two variables increase together, whereas negative correlations indicate inverse relationships.

## 3. Discussion

Mangrove plants face changing salinity levels, tidal flooding, nutrient stress, and oxidative damage, and the microbiota associated with them could provide a precious source of microbial species with combined stress-tolerant characteristics. In the present study, in a stepwise screening, three functionally diverse endophytes, i.e., ACR52, EAL318, and EAR195, were selected for validation in peanut in a greenhouse, both individually and as a consortium of three (C3). A GUS-tagged reporter-based assay revealed that these three mangrove endophytes were capable of entering and colonizing the internal parts of non-host peanut seedlings despite originating from a taxonomically distinct plant species. Cross-host colonization has been demonstrated, so these isolates can be tested for their ability to promote peanut growth, which aligns well with previous findings that mangrove-derived endophytes can establish and grow in non-native cereal hosts under salted conditions [16,17,25].

The combined effects of physiological and biochemical traits clearly revealed that salt tolerance in peanut through endophytic bacteria has primarily resulted from coordinated preservation of water relations, photosynthetic pigments, biomass, antioxidant capacity, osmotic adjustment, and reproductive development. Salinity often reduces plant tissue hydration and chlorophyll stability while simultaneously increasing ROS and lipid peroxidation of membrane lipids. Treatments with the mangrove endophytic bacterial strains preserved better leaf water content and chlorophyll than the control, and at the same time showed better vegetative and reproductive performance; meanwhile, lower H_2_O_2_ and MDA levels indicated less oxidative damage. This scenario aligns with the findings of a recent study on peanuts, in which endophytes of a salt-tolerant type enhanced germination, biomass, and antioxidant activity, decreased MDA accumulation, and regulated osmotic- and defense-related pathways under salinity stress [20].

Among the individual endophytes, ACR52-inoculated peanut plants exhibited the strongest responses, including maintaining higher seed germination, retaining chlorophyll content, conserving relative water content, maintaining nodule number, and maintaining overall plant growth stability under salt-induced stress. The endophyte’s greater capacity to release available phosphate, either in inorganic or organic forms, is evident from the isolate’s high phosphate-solubilizing capacity (unpublished data). Phosphorus is often the limiting element in saline soils, and a sufficient amount of this nutrient in the plant supports root growth, energy metabolism, and photosynthesis. The maintenance of leaf pigments and the yield-related characteristics in ACR52-inoculated peanuts indicate that ACR52 most likely protected the plant from salinity-induced functional limitations rather than boosting antioxidant activity to the highest possible level.

The results obtained for ACR52 align with findings from genus-level research, which reported that salt-tolerant *Klebsiella* isolates can improve crop growth by enhancing IAA synthesis, increasing nutrient availability, promoting nitrogen fixation, and improving crop physiological properties under salinity stress. A *Klebsiella* isolate increased the salinity tolerance and growth of oat seedlings. The use of a halotolerant *K. pneumoniae* strain in rice showed nitrogen-fixing and growth-promoting properties under salt stress [26,27]. However, these results should be mainly interpreted at the genus or functional level, as *Klebsiella quasipneumoniae* subsp. *similipneumoniae* was the strain to which ACR52 belonged, and the precise mechanisms acting in the ACR52 strain remain unknown at the genome or transcriptome level.

EAL318, a *Bacillus subtilis* strain, differed in its function when compared to the other two strains. The EAL318-inoculated peanut plants exhibited better root systems. These plants maintained better root dry mass and simultaneously showed strong SOD and GR activity under severe salinity conditions. SOD dismutates superoxide radicals into H_2_O_2_ and O_2_, and peroxide-scavenging enzymes (CAT, APX, and POD), supported by the GR-dependent glutathione system, then remove the H_2_O_2_. Thus, the high SOD and GR activities observed in EAL318-inoculated plants may reflect rapid superoxide removal coupled with maintenance of the cellular redox balance.

This interpretation is consistent with earlier studies showing that salt-tolerant *Bacillus* strains can maintain better root systems, chlorophyll content, antioxidant defense, and ion homeostasis in rice and wheat. For example, rice inoculation with *B. subtilis* has not only enhanced photosynthetic parameters and antioxidant metabolism, but also Na^+^/K^+^ regulation under salt stress. In contrast, halotolerant *Bacillus* isolates have been reported to promote root growth and physiological traits [28,29]. Since the current study did not measure tissue Na^+^/K^+^ levels or transporter expression, it is very likely that ion homeostasis mechanisms remain a biologically plausible explanation, as supported by previous studies, but the direct effect of EAL318 on ion homeostasis has yet to be confirmed by measuring tissue Na^+^/K^+^ concentrations, ion-transporter expression, and ion-partitioning mechanisms. As such, the findings here provide strong supporting evidence for the hypothesis that EAL318 is key to maintaining root biomass and oxidative stress tolerance through the antioxidant activities of the SOD and GR systems [28,30].

EAR195 exhibited an unusual combination of features. Besides high photosynthesis and strong root production, EAR195 maintained relatively higher leaf number and chlorophyll a under severe salinity and accumulated high levels of antioxidants and soluble sugars while maintaining reproductive output. The observed EAR195 response is consistent with its in vitro PGP phenotype; however, the involvement of IAA, siderophores, or other molecular pathways was not directly demonstrated in planta. It was probably the IAA that supported the plant’s growth from root to shoot. At the same time, a siderophore was a means for bacteria to make iron available in the soil and indirectly support the synthesis of chlorophyll and redox metabolism. Increased soluble sugars probably helped with osmotic adjustment, carbon provision, and the stability of the cell membrane structure under salt stress.

Compared with *Bacillus*, only a few studies have examined the beneficial effects of *Proteus mirabilis* on plants under saline conditions. Despite this, stress-resistant *P. mirabilis* strains can produce IAA and ACC deaminase to reduce the stress hormone ethylene and solubilize nutrients, making them available in the rhizosphere, thereby increasing wheat growth under drought stress and reducing oxidative injury in maize under metal exposure [31,32].

The research findings further underscore that, in addition to salinity, *P. mirabilis* may help plants overcome various abiotic stresses. However, these studies do not provide evidence that the same mechanism has been responsible for the response of EAR195 to the salinity treatment. For this reason, the positive impacts of EAR195 observed in this paper are first and foremost attributed to altered IAA and siderophore levels, shoot biomass, chlorophyll, APX and GR activities, soluble sugars, and reproductive performance; however, the molecular pathways remain to be experimentally verified.

The three-strain consortium C3 (ACR52, EAL318, and EAR195) provided the greatest overall protection against salinity, although it did not achieve the highest scores across all measured parameters. C3 provided one of the broadest greenhouse-stage responses, particularly through the maintenance of shoot and leaf biomass, osmotic adjustment, nodulation, flowering, and coordinated antioxidant defense. The concurrent increase in peroxide-detoxification enzymes and the reduction in an oxidative-damage indicator suggest that this consortium induced a more balanced antioxidant response than treatments in which a single component of the antioxidant system was maximized.

These results indicate that the consortium protected plants against oxidative stress by inducing antioxidant enzymes while minimizing osmotic damage through osmotic adjustments, carbon reserve preservation, and plant growth promotion. The overall performance of C3 can be explained through functional complementarity among the constituent strains, where ACR52 significantly contributed to chlorophyll maintenance, crop water status, and reproductive stability while EAL318 largely improved root growth and SOD- and GR-mediated antioxidative defenses and EAR195 enhanced shoot biomass, APX- and GR-mediated responses, soluble-sugar accumulation, and reproductive performance.

The combination of functionally distinct isolates addressed a broader spectrum of biochemical and physiological plant stress responses than individual strains. Still, the present study shows a complementary triple-combination effect rather than a proven synergistic effect, since the consortium response was not statistically compared with the anticipated additive effects of ACR52, EAL318, and EAR195 individually. Similar outcomes were obtained when mangrove microbial consortia were selected to improve rice salt tolerance by modulating soluble sugars, proline, lipid peroxidation, ionic balance, and stress-inducible gene expression [16]. All in all, the application of native-compatible strains with divergent functional traits may result in a broader range of stress protection, taking into account inoculum ratios, host crop, and stress severity, but consortium performance is, in turn, dependent on the compatibility traits of the component strains.

### 3.1. Integration of Water Status, Photosynthetic Pigments, Antioxidant Defense and Osmotic Adjustment

Taken together, the biochemical and physiological data suggest that maintaining leaf water status, photosynthetic pigment concentrations, and biomass was the key factor underpinning bacterial-induced peanut salinity tolerance. Chlorophyll a and total chlorophyll content were both positively correlated with growth and yield-related attributes, implying that the maintenance of the photosynthetic system played a role in dictating reproductive output. Both relative water content and fresh-biomass traits were also positively correlated with plant growth and production, suggesting cellular hydration and retention of functional leaf tissue were important for salinity tolerance. Photosynthetic rate was not directly quantified, but the higher chlorophyll and fresh-biomass content recorded in the inoculated peanut suggest their photosynthetic systems were less affected than those of their uninoculated counterparts.

Conversely, H_2_O_2_ content was negatively correlated with biomass and yield attributes, indicating that peroxide accumulation is a major constraint on peanut production under salinity. Malondialdehyde concentration reflects increased membrane lipid peroxidation, while excessive H_2_O_2_ accumulation can signal an imbalance in ROS production and scavenging. Antioxidant enzyme activity levels should be viewed against the background of increased oxidative damage rather than separately. The SOD, APX, CAT, POD, and GR data indicate either a successful defense against oxidative stress or that these plants experienced a greater oxidative challenge than the other treatments. The presence of high CAT and POD activity in the C3 consortium, together with low H_2_O_2_ and malondialdehyde levels under high salinity, provides the strongest argument for successful oxidative defense. By comparison, EAL318 predominantly enhanced the activity of the SOD and GR components of the antioxidant network, but little APX, whereas EAR195 preferentially stimulated APX and GR.

Compatible solutes (proline) and carbohydrate reserves provide two additional components to stress acclimation. Proline has been implicated in osmotic regulation, stabilization of both proteins and membranes, and re-establishment of cellular redox homeostasis. Water-soluble sugars (predominantly the hexose sugars glucose and fructose) also function in osmolyte regulation, as well as functioning as key signaling molecules and internal substrates. The retention of starch reserves reported in consortium C3-treated plants may further act to promote ongoing growth and facilitate subsequent recovery processes. The C3 Consortium also exhibited the highest proline content and starch retention, indicating an ability to regulate osmotic potential and conserve carbon reserves. In reality, EAR195-treated plants had relatively high levels of water-soluble sugars, indicating that this consortium may have affected osmotic regulation and energy metabolism to a greater extent than the other treatments.

The fact that proline, soluble sugars, amino acids, and starch show variable correlations with yield suggests that merely accumulating a single osmolyte cannot be a reliable indicator of salt tolerance. Instead, successful salt tolerance depends on the simultaneous maintenance of tissue water content and chlorophyll, biomass, and the development of reproductive organs, as well as on the sufficient osmotic adjustment and restriction of oxidative damage. This means that the most effective bacterial treatments are those that combine physiological maintenance with antioxidant and metabolic protection, without maximizing a single stress-responsive trait. Such a combination of tolerance mechanisms is consistent with previously reported cases in which osmolyte accumulation, carbohydrate regulation, and antioxidant regulation have simultaneously contributed to bacterial-mediated salinity tolerance in crop plants [33,34].

### 3.2. Nodulation, Flowering, and Yield Retention

Salinity negatively affects legume nodulation through the disruption of rhizobial survival, root-hair infection, nodule initiation, and nitrogen fixation. In the current experiment, the ACR52 and C3 consortium retained more nodules and flowers under salinity, but ACR52 and EAR195 were more effective in preserving pod yield, seed DW, and harvest index at 500 mM NaCl. These results reflect the differential potential of the single strains for vegetative protection and for translating such tolerance into reproductive output.

The support for the positive correlations among chlorophyll, vegetative biomass, pod number, seed number, 100-seed weight, and harvest index indicates a need to maintain photosynthetic sources and an active vegetative setup to support reproductive output. However, increased nodule number is not an indication of increased nitrogen fixation; without validation of nodular activity, leghemoglobin levels, nitrogenase activity, and the relative abundance of rhizobium, it is not possible to determine whether nitrogen fixation has increased. Enhancement of nodulation and yield-related variables is supported by the in vivo response. Still, the symbiosis cannot be confidently interpreted as involving increased nitrogen fixation [35,36].

### 3.3. Limitations of the Present Study and Future Validation

Despite consistent physiological, biochemical, and reproductive responses following bacterial inoculation, several limitations must be considered when interpreting the present conclusions. First, tissue Na^+^ and K^+^ concentrations, ion partitioning, Na^+^/K^+^ ratios, photosynthetic gas exchange, or the expression of ion-transporter, antioxidant, and stress-responsive genes were not quantified in the present study. Therefore, mechanisms involving ion homeostasis, hormone signaling, enhanced carbon assimilation, and induced systemic tolerance should be interpreted as biologically plausible mechanisms, supported by earlier work, rather than as mechanisms proven by this study. The observed increases in chlorophyll, relative water content, antioxidant enzymes, osmolytes, MDA, and H_2_O_2_ lend support to the concept of enhanced physiological and redox regulation. Still, more ionomic, transcriptomic, and photosynthetic data are needed to determine the molecular mechanisms involved.

Secondly, the extreme greenhouse treatments of 300 and 500 mM NaCl, although useful for categorizing the stress-mitigating ability of the bacterial treatments, are unlikely to reflect inoculant performance in more complex saline agricultural soils, where salinity is induced more slowly and interacts with other edaphic factors (including native soil microbiota). Research should be undertaken on the inoculants in multi-season field trials on graded soil EC levels in non-sterile soils before any field-use recommendations are made.

Lastly, the use of 16S rRNA gene sequencing provided information on the closest taxonomic relationships of the isolates, but did not provide sufficient strain-specific information to verify the identity, genome stability, or biosafety of the bio-inoculants. Verification of strain identity and genome stability for biosafety screening of plant growth promotion, at least under in vitro conditions, as well as for antimicrobial resistance, mobile elements, and potential virulence, should be performed through whole-genome sequencing of the isolates before field trials with the inoculants. Phenotypic biosafety testing should also be performed before field trials on those isolates, whose closest taxonomic position may suggest prior, strain-specific hazards. The inoculants in the current study are best viewed as novel bio-inoculant candidates at this stage rather than ready-to-apply commercial inoculants.

Despite these limitations, the findings show that the three mangrove-derived endophytes provided distinct yet complementary protective effects against salt stress in peanut. ACR52 was mainly related to germination, chlorophyll maintenance, plant water status, and reproductive stability; EAL318 was mainly related to root-system maintenance and SOD/GR-associated antioxidant defense; and EAR195 was mainly related to shoot biomass, soluble-carbon adjustment, and APX/GR-associated responses. Their combined use in C3 has provided the widest synchronization of physiological maintenance, osmotic adjustment, and oxidative-stress mitigation. The results of this research will provide a solid foundation for further studies on genome-based biosafety assessment and validation of effects under agronomically realistic saline soil conditions.

## 4. Materials and Methods

### 4.1. Collection and Isolation of Halotolerant Endophytic Bacteria from Mangrove Plants

Two mangrove species, *Aegiceras corniculatum* and *Excoecaria agallocha*, were identified from mangrove ecosystems along the Duggirajapatnam and Krishnapatnam shorelines in Sri Potti Sriramulu Nellore District, Andhra Pradesh, India (14°01′0″–14°02′30″ N; 80°08′0″–80°09′30″ E), based on the previously reported literature [37]. Samples including leaves, stems, roots, rhizospheric soil, and sediments were collected from both species at different locations following the procedure described by [38].

Endophytic bacterial isolates were obtained from root and leaf tissues using modified protocols described by [39]. Briefly, crushed tissues were serially diluted up to 10^−4^ and spread on nutrient agar plates prepared according to [40]. Plates were incubated at 28–30 °C for 48 h. Morphologically distinct colonies were selected and purified by repeated streaking on nutrient agar supplemented with Bavistin (10 µg mL^−1^) (Crystal Crop Protection Pvt. Ltd, India) to prevent fungal contamination. Purified isolates were preserved as glycerol stocks and maintained at 4 °C for further use.

Prior to plant experimentation, bacterial isolates were subjected to preliminary screening for salinity tolerance and basic morphological and molecular identification.

### 4.2. Salt Stress Effects on Germination of Endophyte-Inoculated Peanut (Cv. JL24)

A factorial laboratory experiment was conducted to evaluate the effects of six mangrove-derived halotolerant endophytic bacterial isolates on the germination and early growth of peanut (*Arachis hypogaea* L. cv. JL24) (procured from the Regional Agriculture Research Station (RARS), Tirupati, India) under salinity. The isolates evaluated were ACR52 (*Klebsiella quasipneumoniae* subsp. *similipneumoniae*; GenBank accession PV657114), EAR173 (*Proteus mirabilis*; PV660264), EAR191 (*Sinocapsa zengkensis*; PV661994), EAR195 (*Proteus mirabilis*; PV656438), EAL318 (*Bacillus subtilis*; PV656428), and ACL463 (*Enterobacter* sp.). These isolates had previously been shortlisted based on their halotolerance and plant growth-promoting characteristics, including phytohormone production and nutrient-solubilization potential. The experiment consisted of eight bacterial treatments: six individual isolates, a six-strain consortium designated C6, and an uninoculated control. C6 comprised ACR52, EAR173, EAR191, EAR195, EAL318, and ACL463 mixed in a 1:1:1:1:1:1 ratio. For the preparation of C6, the individual cultures were adjusted to comparable cell densities before mixing, and the final total bacterial density of the combined inoculum was standardized to approximately (10^8^ CFU mL^−1^); thus, (10^8^ CFU mL^−1^) refers to the total viable bacterial population in C6 rather than (10^8^ CFU mL^−1^) of each component strain. This compositionally defined six-strain consortium (C6) was used exclusively in the laboratory seedling-stage experiment and was distinct from the three-strain consortium C3 used in the subsequent greenhouse experiment.

The sterilized seeds were then treated with bacterial suspensions (10^8^ CFU mL^−1^) by shaking at 120 rpm for 2 h in darkness, either individually or as a bacterial consortium, and subsequently air-dried under laminar airflow (Technico Laboratories Pvt Ltd, India) [41,42]. To verify the effectiveness of surface sterilization, representative surface-sterilized peanut seeds were subjected to a seed-imprint test before bacterial inoculation. Each sterilized seed was gently pressed and rolled across the surface of nutrient agar plates to transfer any remaining culturable surface-associated microorganisms into the medium. The imprint plates were incubated at 28 °C for 48 h. Simultaneously, a final rinse of surface-sterilized water was plated on LB media, and the plates were incubated at 28 °C for 48 h. The absence of bacterial or fungal colony growth on the imprint plates was considered to be evidence of the effective removal of culturable epiphytic microorganisms from the seed’s surface. The test was performed using three independent seed batches. We employed both bacterial inoculation and salinity treatments as factors, arranged in a factorial manner, to generate all possible experimental treatment combinations. For every treatment combination, 10 surface-sterilized seeds were added to each sterile Petri dish, with a sterile filter paper at the bottom. Each treatment (NaCl concentrations: 0 mM L^−1^ (control), 50 mM L^−1^, 100 mM L^−1^, 150 mM L^−1^, 200 mM L^−1^, and 250 mM L^−1^) combination was performed in three independent Petri dishes, constituting three biological replicates. The Petri dish itself was an experimental unit, but the individual seeds and seedlings within it were considered sub-samples. Seeds were germinated under controlled laboratory conditions (25 °C, 16/8 h light/dark photoperiod, and 70% relative humidity). Germination was recorded on Days 1, 4, and 11 after initiation of the experiment. After the final count, the germination percentage was calculated according to the formula described by [43].

The germination percentage (GP) was calculated for each Petri dish using the following formula:

$$GP(\%)=\frac{Total\;number\;of\;germinated\;seeds}{Total\;No\;of\;seeds\;tested}\times 100$$

Seedling vigor index (VI) was calculated according to the method described by [44] using the following formula [45]:

VI = (RL + SL) × GP

where RL represents root length and SL represents shoot length.

Seedling morphological parameters, including root length and shoot length, were measured on the 10th day in each dish for all the seedlings, and the average was calculated for each parameter. Plant height was also recorded at the same time point. Biomass-related parameters, including fresh weight, dry weight, and total seedling biomass, were determined following the procedure [46]. We used dish-level mean values for the statistical analysis.

### 4.3. Reporter-Based Histochemical Assessment of Endophytic Bacterial Colonization in Peanut

ACR52, EAR173, EAR191, EAR195, EAL318, and ACL463 were selected for assessment of their ability to colonize the non-native host peanut (*Arachis hypogaea* L. cv. JL24). The isolates were tagged with the β-glucuronidase reporter gene, *gusA*, to enable histochemical visualization of their spatial distribution within peanut tissues. The pCAM111 plasmid vector carrying the *gusA* reporter gene was kindly provided as a gift by Dr. Birgit Mitter, Austrian Institute of Technology (AIT), Vienna, Austria. The donor plasmid pCAM111 carrying the *gusA* reporter gene was mobilized into the recipient bacterial isolates by conjugation following the procedure described by [47] with appropriate modifications. Successful acquisition and expression of the reporter construct were confirmed by the development of blue coloration following exposure to X-Gluc substrate. Reporter-tagged derivatives were subsequently cultured in LB media. To determine the colonization efficacy of ACR52-*gusA*, EAR 173-*gusA*, EAR191-*gusA*, EAR195-*gusA*, EAL 318-*gusA*, and ACL463-*gusA*, the six bacterial strains were initially grown in LB medium at 30 °C until the optical density (OD) at 600 nm reached 0.6. The culture was centrifuged (5804 R, Eppendorf, Hamburg, Germany) at 8000 rpm for 10 min, and the pellet was resuspended in sterile water. Seeds of the non-host plants *Arachis hypogaea* (L.) (cv. JL24) were surface-sterilized with 2% sodium hypochlorite for 40 s, followed by 4–5 thorough rinses with double-distilled water to remove the residual disinfectant. Uniformly sized surface-sterilized seeds were incubated in the respective bacterial suspension, and non-inoculated seeds were incubated in sterile water for 10 h. Furthermore, the inoculated seeds were placed on moistened Whatman filter paper in sterile Petri dishes and incubated in a growth chamber maintained at 25 °C with a 16/8 h photoperiod. Ten-day-old seedlings were used for the GUS staining and microscopic studies. The presence of transconjugants was confirmed using the X-gluc staining solution [48]. After 24 h of incubation in X-gluc solution, the seedlings were washed with sterile water and dipped in 70% ethanol for 3–4 h to better visualize the GUS staining. Then, the hand-cut transverse sections (TS) of root, stem, and leaf tissues were photographed under a light microscope (CX-23 Binocular LED Microscope, Olympus Corporation, Tokyo, Japan).

### 4.4. Halotolerant Plant Growth-Promoting Bacteria Effect on Growth and Yield of Peanut (Cv. JL24) Under Salinity Stress

For the greenhouse experiment, three halotolerant endophytic bacteria, i.e., EAL318, EAR195, and ACR52 strains, were selected to evaluate their ability to enhance salinity tolerance in the dicotyledonous non-host crop peanut (*Arachis hypogaea*, cv. JL24).

Twenty uniform-sized surface-sterilized peanut seeds (cv. JL24; salt-sensitive) for each treatment were inoculated with individual (EAL 318, EAR 195, and ACR52) bacterial suspensions (10^8^ CFU mL^−1^) or with a bacterial consortium C3 (ACR52 + EAL318 + EAR195). The bacterial suspensions were prepared in nutrient broth (M002, HiMedia Laboratories Pvt. Ltd., India) containing peptone (0.5% *w*/*v*), beef extract (0.3% *w*/*v*), and NaCl (0.5% *w*/*v*) at pH 7.0, supplemented with 1% jaggery and 0.4% Carboxymethyl cellulose (CMC) (RM10844, HiMedia Laboratories Pvt. Ltd., India) as an adhesive. The inoculated seeds were incubated and subsequently air-dried under laminar airflow following previously described procedures [41,42].

Both endophyte-inoculated and mock-treated seeds (uniform in size and number) were initially placed on sterile filter paper in Petri dishes in triplicate for pre-germination. Subsequently, the pre-germinated (3 seedlings each) seeds were transplanted into pots containing 6 kg of sterilized soil mixture (black soil:redsoil:sand; 2:1:1). The greenhouse experiment was carried out as a factorial design with five inoculation treatments—uninoculated control ACR52, EAL318, EAR195, and the three-strain consortium C3—and three NaCl levels: 0, 300, and 500 mM. The three-strain consortium C3 comprised ACR52, EAL318, and EAR195 mixed in an equal ratio (1:1:1). The plants were grown in the greenhouse at Yogi Vemana University, Kadapa, under open conditions at approximately 25 °C, with a 16/8 h photoperiod, and were irrigated weekly. Salinity treatments were gradually applied at the 40th and 60th days after transplantation into pots for vegetative stress and reproductive stage, respectively, by adding 700 mL of 150 mM NaCl every other day until they reached the target concentrations, as monitored by EC 25 dS/m (300 mM NaCl) and 42 dS/m (500 mM NaCl) (CM180, Elico, India). Control pots received an equal volume of distilled water. The pots were randomly organized in the greenhouse, and their positions were changed periodically to reduce the effects of position. Each pot was selected as the experimental unit, as the inoculation and salinity treatments were independently applied at the pot level. A separate set of pots was maintained for reproductive traits.

### 4.5. Measurement of Morphological and Physiological Traits

At 65 days post-transplantation, peanut plants (cv. JL24) from both inoculated and control treatments were evaluated for morphological, biomass, and physiological parameters under different NaCl concentrations. For each inoculation, NaCl combination, 9 plants were selected from 3 pots (3 plants within each pot considered as sub-samples) (n = 3 biological replicates) to measure the root length, shoot length, plant height, number of leaves, leaf fresh and dry weight, specific leaf area, and total biomass. Physiological traits, including photosynthetic pigments and relative water content (RWC), were also assessed. RWC was determined by measuring the fresh weight, turgid weight, and dry weight of leaf samples collected from inoculated and uninoculated plants. All measurements were conducted following the method of [49]:

RWC = RWC (%) = (FW − DW)/(TW − DW) × 100

Chlorophyll content was determined following the method of [50] using the acetone extraction method. Leaf pigments were extracted with 80% acetone, and absorbance was measured at 645 nm and 663 nm using a UV–Vis spectrophotometer (UV-1800 UV-Visible Spectrophotometer, Shimadzu Corporation, Japan), with 80% acetone as the blank.

Total chlorophyll, chlorophyll a, and chlorophyll b contents were calculated using standard equations and expressed as mg g^−1^ of fresh tissue:

Total chlorophyll (mg g^−1^ FW) = [20.2 (A_645_) + 8.02 (A_663_)] × *V*/(1000 × *W*)

Chlorophyll *a* (mg g^−1^ FW) = [12.7 (A_663_) − 2.69 (A_645_)] × *V*/(1000 × *W*)

Chlorophyll *b* (mg g^−1^ FW) = [22.9 (A_645_) − 4.68 (A_663_)] × *V*/(1000 × *W*)

where A_645_ and A_663_ are the absorbances at 645 and 663 nm, *V* is the final volume of the 80% acetone extract (mL), and *W* is the fresh weight of the leaf sample (g).

### 4.6. Effect of Endophytic Bacteria on Antioxidant Enzymes in Peanut Seedlings Under Salinity

At 65 days post-transplantation, for each inoculation and NaCl combination, 9 plants were selected from 3 pots (3 plants within each pot considered as sub-samples) (n = 3 biological replicates), and leaf and root samples were collected separately to assess antioxidative enzyme activities and related biochemical parameters following the methods of [51].

Total proteins for antioxidant enzyme assays were extracted from inoculated and control peanut leaves using phosphate buffer as described by [51], with minor modifications. Extracts were centrifuged at 12,000 rpm for 20 min at 4 °C, and the supernatant was stored at −20 °C for biochemical analyses. Protein content was quantified using the Biuret method [52], with BSA as the standard. Briefly, 0.2 mL of protein extract was mixed with 5 mL Biuret reagent, incubated at 37 °C for 10 min, and the resulting violet color, due to the reaction of alkaline copper sulfate with peptide bonds, was measured at 540 nm using a double-beam spectrophotometer (Systronics 2203). Protein concentrations were determined from a BSA standard curve. Catalase (CAT) activity was measured as the decomposition of H_2_O_2_ into water and oxygen, monitored by the decrease in absorbance at 240 nm [53], which is directly proportional to enzyme activity. Glutathione reductase (GR) activity was assayed following [54], where GSSG is reduced to GSH using NADPH. The reduced GSH reacts with DTNB to form a colored product, which was measured at 412 nm, and enzyme activity is expressed as U (nmol NADPH oxidized min^−1^ mg^−1^ FW). The decrease in absorbance at 290 nm is proportional to GR activity. Superoxide dismutase (SOD) activity was determined by measuring the inhibition of NBT reduction, in which SOD competes with NBT for superoxide radicals, thereby reducing formazan formation; the decrease in formazan formation reflects SOD activity [55]. Peroxidase activity was measured by the oxidation of guaiacol by H_2_O_2_, resulting in the formation of a brown-colored product, and POD content was measured using a spectrophotometer, taking the absorbance values at 470 nm. Malondialdehyde (MDA) content, an indicator of lipid peroxidation and oxidative damage, was measured using the TBA assay, where elevated MDA forms a red-colored complex. Hydrogen peroxide (H_2_O_2_) content was estimated using diaminobenzidine (DAB) staining, producing a dark brown precipitate quantified at 390 nm [54]. Ascorbate peroxidase (APX) activity was determined by monitoring the oxidation of ascorbate during the reduction of H_2_O_2_ to water, with a decrease in absorbance at 290 nm reflecting enzyme activity [56].

Sugars were estimated in inoculated and non-inoculated peanut leaf samples by preparing leaf extracts with 80% ethanol. After centrifugation at 12,000 rpm for 15 min at 4 °C, the supernatant was stored at −20 °C for further biochemical analyses. The final ethanolic extract was utilized for the estimation of total soluble sugars [57]. The determination of phenolic content was measured as per [58]. Using Folin–Ciocalteu reagent (a mixture of phosphomolybdate and Phosphotungstate), phenolic compounds form a molybdenum-tungsten blue-colored complex, and the content is calculated using a standard curve prepared with gallic acid. Proline estimation [30] was measured using the ninhydrin reagent, which reacts with proline to form a red-colored complex, resulting in the formation of a chromophore, and then the proline content is calculated using a standard curve prepared with proline. Free amino acid content [59] can be calculated with the ninhydrin reagent, which reacts with the free amino acids to form a purple-colored complex. The reaction involves the oxidative deamination of amino acids, resulting in the formation of an aldehyde and the release of ammonia. The free amino acid content can then be calculated using a standard curve prepared with glycine.

Each spectrophotometric assay was replicated technically. For each extract, the three values from a technical triplicate were combined and averaged to yield a single value for that biological replicate. Technical triplicate readings were for the analytical precision assessment only, and were not regarded as independent samples in the statistical analysis.

### 4.7. Measurement of Reproductive Traits in Peanut at Harvest (110 Days After Transplanting)

At 110 days of post-transplantation, for each inoculation, NaCl combination, 9 plants were selected from 3 pots (3 plants within each pot considered as sub-samples) (n = 3 biological replicates), and several reproductive traits, including Pod Number, Pod Yield, Number of Seeds, Seed Fresh Weight, Seed Dry Weight, 100 Seed Weight, Haulm Fresh Weight, Dry Weight, and Biomass were measured. The fresh and dry weights and total biomass of the haulm were measured using the method of [60].

Harvest index (HI) was calculated according to [61] and is expressed as a percentage. The harvest index of all Peanut(cv JL24) plants was calculated using the following formula:

Harvest Index (%) =Pod yield/total biomass of plant × 100

### 4.8. Statistical Analysis

Peanut germination and seedling tests were carried out in the laboratory. The Petri dish was the experimental unit for statistical analysis, whereas for greenhouse experiments, each separately treated pot was the experimental unit. Data from the seedling and plants were taken from the same experimental unit and averaged out before running statistical analyses. Morphological, physiological, biochemical, and yield data were analyzed using a two-way analysis of variance, with bacterial inoculation and NaCl concentration as fixed effects. Wherever significant effects occurred, the respective treatment means were compared using Tukey’s HSD test at *p* < 0.05. The normality of the data was checked using the Shapiro–Wilk test, and Levene’s test was used to assess assumptions before running parametric tests such as *t*-tests or ANOVA. These tests were used to verify whether the data follow a normal distribution and whether the variances are equal across groups. The germination variables observed repeatedly for each Petri dish at Days 1, 4, and 11 were statistically analyzed using a two-way ANOVA for each observation day, selecting only the test actually performed. Each principal component and Pearson correlation analysis was performed with XLSTAT Version 2010.5.05 [62], and the findings were depicted using R software (R-4.6.1 for Windows) packages to evaluate the strength and direction of the linear relationship between two continuous, normally distributed variables. The observational unit and n. for each multivariate analysis are indicated in the respective figure legends.

## 5. Conclusions

Endophytic bacteria from mangrove plants reduced the adverse effects of salinity on peanut cv. JL24, but the type and level of protection also varied between isolates. For instance, ACR52 was related mainly to seed germination, chlorophyll retention, and reproduction performance; EAL318 exhibited a better preservation of roots and also a higher level of antioxidant activity (SOD/GR); and EAR195 was responsible for the shoot biomass development, soluble sugar adjustment, and also the protection provided by the APX/GR antioxidant system. The combination of three strains, C3, offered the most diverse responses, combining maintenance of physiological processes, osmotic adjustments, and protection against oxidative damage. Based on the reporter assay, it was established that peanut seedlings are colonized early by the microorganisms; however, the questions of prolonged persistence, how the microorganisms affect ion balance, strain-level biosafety, and their effectiveness under realistic field conditions remain to be addressed. The results offer theoretical support for microbial formulation, genome-based safety analysis, and field assessment.

## Data Availability

The original contributions presented in this study are included in the article/Supplementary Material. Further inquiries can be directed to the corresponding author.

## Supplementary Materials

The following supporting information can be downloaded at https://www.mdpi.com/article/10.3390/plants15203218/s1. Figure S1: Fresh Biomass of peanut seedlings under NaCl stress; Figure S2: Dry Biomass of peanut seedlings under NaCl stress; Figure S3: Effects of selected mangrove-derived endophytic bacteria and the three-strain consortium C3 on osmolyte and carbohydrate contents of peanut under salinity; Figure S4: Representative photographs showing the effects of halotolerant endophytic bacterial inoculation on peanut plants during the reproductive stage under NaCl stress; Table S1: Root antioxidant-enzyme activities and oxidative-damage indicators of peanut cv. JL24 under salinity.
