# Supplementary material for: Mangrove-Derived Halotolerant Endophytic Bacteria Confer Strain-Specific and Consortium-Mediated Salt Tolerance in Peanut (Arachis hypogaea L.)
Source: Plants (Basel). 2026 Oct 20;15(20):3218. doi: 10.3390/plants15203218 (PMC13708864; doi:10.3390/plants15203218)
Supplement: Supplementary file 1 [file plants-15-03218-s001.zip › plants-4447330-supplementary.pdf]

SUPPLEMENTARY FIGURES:

Figure S1. Effects of mangrove-derived endophytic bacterial inoculation on seedling

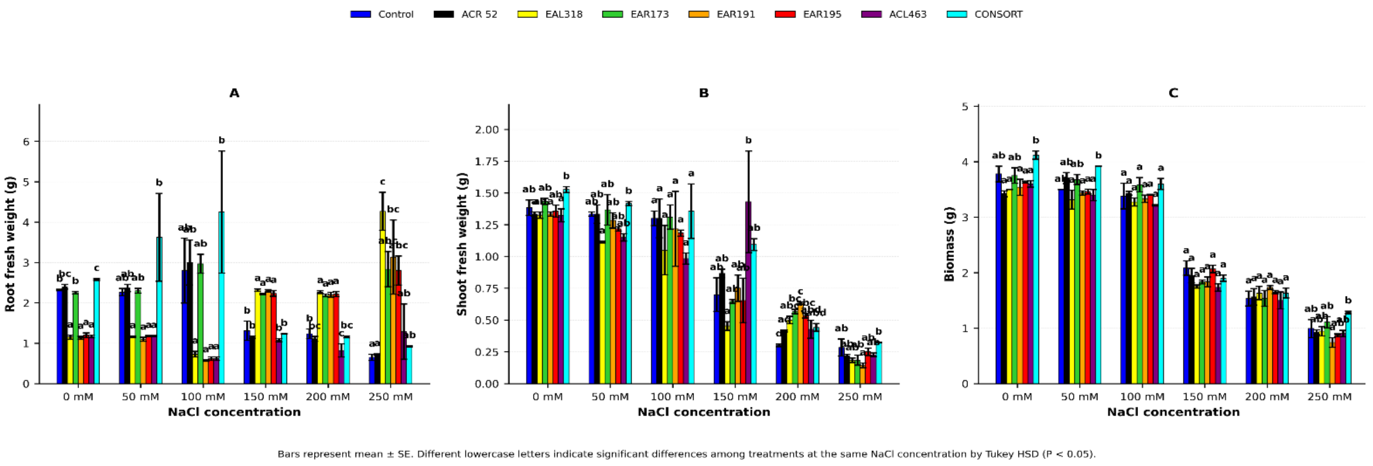

Supplementary Figure S1. Fresh Biomass of peanut seedlings under NaCl stress. Surface-sterilized seeds of peanut cv. JL24 were inoculated individually with ACR52, EAR173, EAR191, EAR195, EAL318 or ACL463, or with the six-strain consortium C6 comprising all six isolates; uninoculated seeds served as the control. Biomass was determined on Day 11th after exposure to 0, 50, 100, 150, 200 and 250 mM NaCl. (A) root fresh weight (B) shoot fresh weight (C) Biomass. Values represent mean ± SE of three independent biological replicates per bacterial treatment × NaCl combination (n=3), with each replicate derived from one independently treated pot. Spectrophotometric measurements were performed in technical triplicate, and the technical readings were averaged to obtain one value for each biological replicate. Data were analysed by two-way ANOVA with bacterial inoculation and NaCl concentration as fixed factors, including their interaction followed by Tukey's multiple-comparison test. Different letters indicate significant differences at $p < 0.05$.

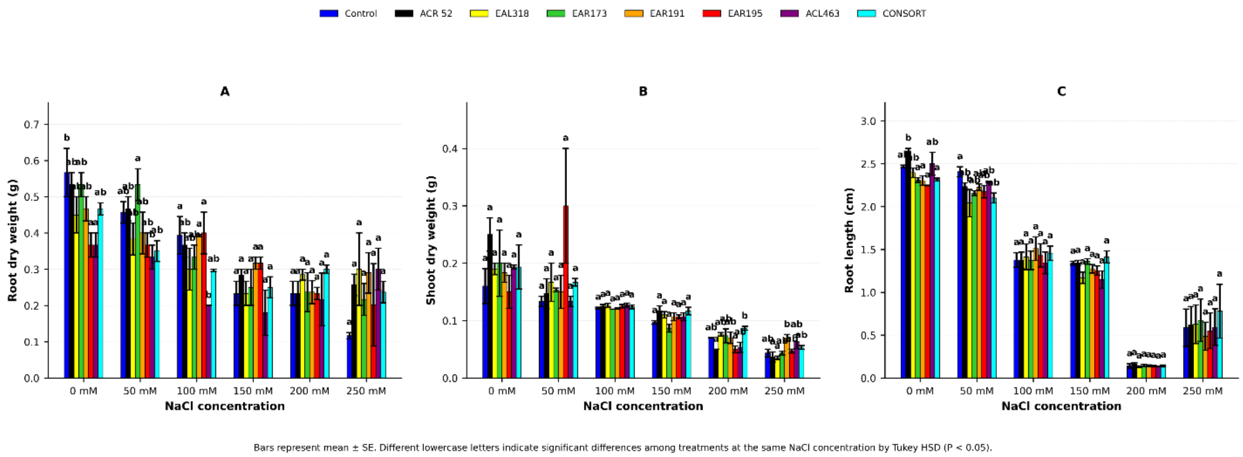

Supplementary Figure S2. Dry Biomass of peanut seedlings under NaCl stress. Surface-sterilized seeds of peanut cv. JL24 were inoculated individually with ACR52, EAR173, EAR191, EAR195, EAL318 or ACL463, or with the six-strain consortium C6 comprising all six isolates; uninoculated seeds served as the control. Biomass was determined on Day 11th after exposure to 0, 50, 100, 150, 200 and 250 mM NaCl. (A) root dry weight (B) shoot dry weight (C) root length. Values represent mean ± SE of three independent biological replicates per bacterial treatment × NaCl combination (n=3), with each replicate derived from one independently treated pot. Spectrophotometric measurements were performed in technical triplicate, and the technical readings were averaged to obtain one value for each biological replicate. Data were analysed by two-way ANOVA with bacterial inoculation and NaCl concentration as fixed factors, including their interaction followed by Tukey's multiple-comparison test. Different letters indicate significant differences at $p < 0.05$.

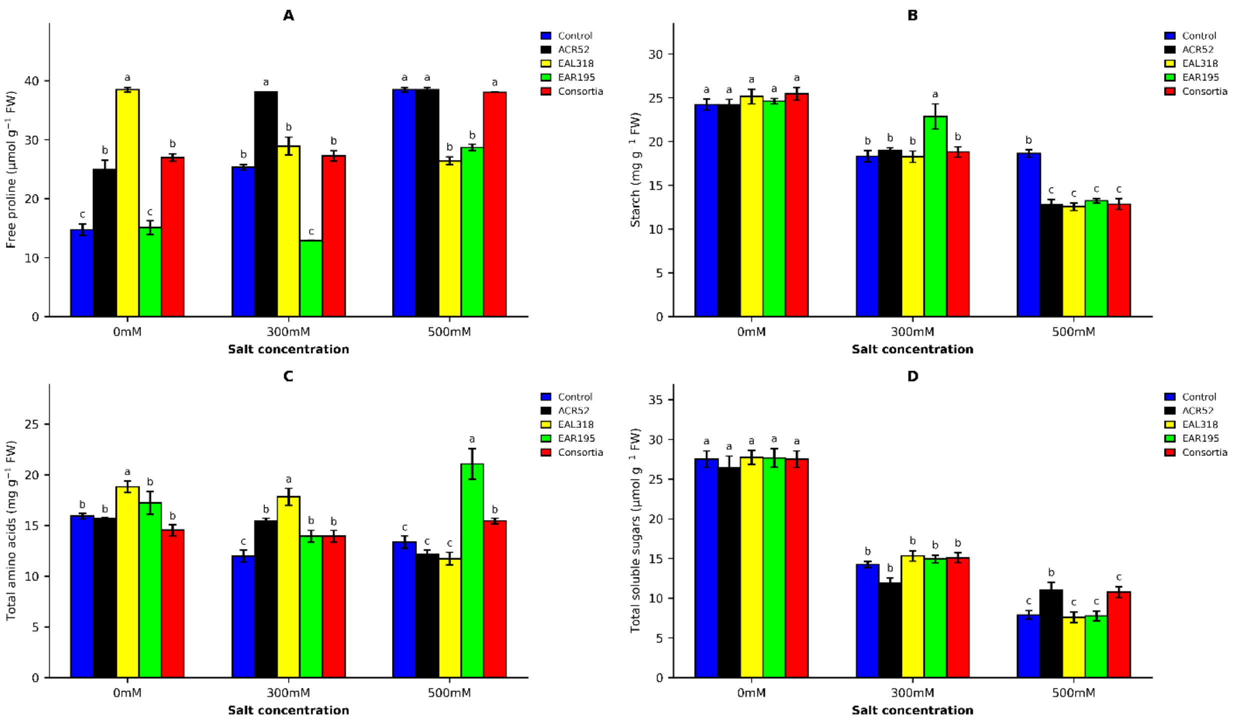

Supplementary Figure S3. Effects of selected mangrove-derived endophytic bacteria and the three-strain consortium C3 on osmolyte and carbohydrate contents of peanut under salinity. Peanut cv. JL24 plants were inoculated with ACR52, EAL318, EAR195 or the three-strain consortium C3 comprising ACR52, EAL318 and EAR195; uninoculated plants served as the control. Plants were exposed to 0, 300 and 500 mM NaCl. The measured biochemical parameters were: (A) Free Proline, (B) starch content, (C) Total amino acid content and (D) Total soluble sugar content. Values represent mean ± SE of three independent biological replicates per bacterial treatment × NaCl combination (n=3), with each replicate derived from one independently treated pot. Spectrophotometric measurements were performed in technical triplicate, and the technical readings were averaged to obtain one value for each biological replicate. Data were analysed by two-way ANOVA with bacterial inoculation and NaCl concentration as fixed factors, including their interaction followed by Tukey's multiple-comparison test. Different letters indicate significant differences at $p < 0.05$.

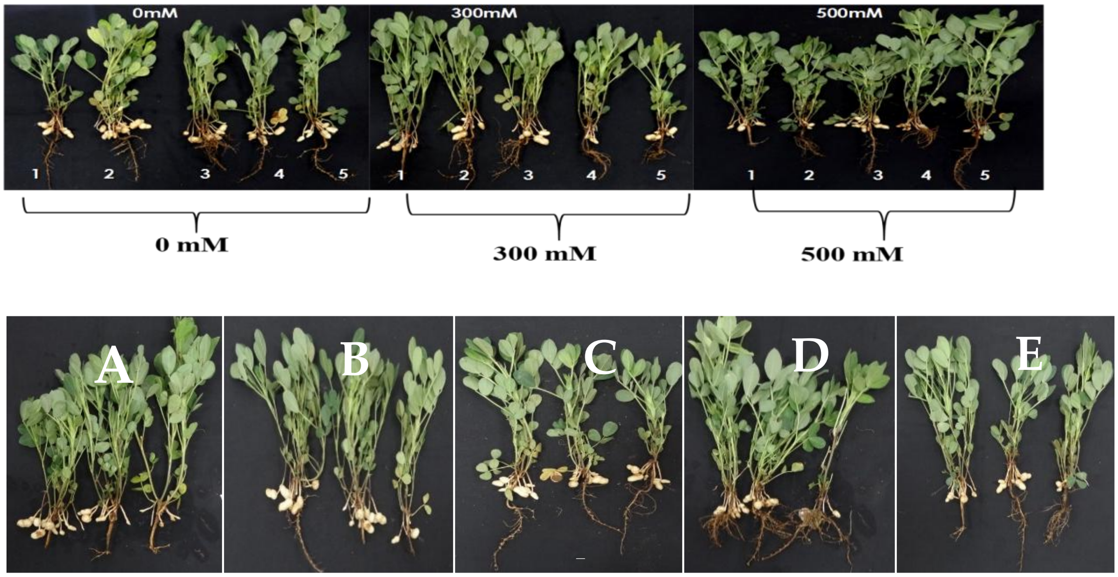

Supplementary Figure S4. Representative photographs showing the effects of halotolerant endophytic bacterial inoculation on peanut plants during the reproductive stage under NaCl stress. Peanut cv. JL24 plants were inoculated with ACR52, EAR195, EAL318 or the three-strain consortium C3 comprising ACR52, EAR195 and EAL318; uninoculated plants served as the control. Plants were grown under 0, 300 and 500 mM NaCl. In panel I, treatments within each salinity level are arranged as: 1, uninoculated control; 2, ACR52; 3, EAR195; 4, EAL318; and 5, C3. In panel II, bacterial treatments are arranged as: (A) uninoculated control, (B) ACR52, (C) EAR195, (D) EAL318 and (E) C3, with 1, 2 and 3 representing 0, 300 and 500 mM NaCl, respectively. The photographs illustrate differences in plant growth and reproductive performance associated with increasing salinity.

Supplementary Table S1. Root antioxidant-enzyme activities and oxidative-damage indicators of peanut cv. JL24 under salinity							
**Treatment**	**NaCl (mM)**	**SOD (μmol.g^{-1}FW)**	**CAT (μmol.g^{-1}FW)**	**APX (μmol.g^{-1}FW)**	**POD (μmol.g^{-1}FW)**	**H_2O_2 (μmol.g^{-1}FW)**	**MDA (μmol.g^{-1}FW)**
Control	0	15.00 ± 0.30	15.22 ± 0.34	28.51 ± 0.33	12.88 ± 0.54	1.94 ± 0.06	2.78 ± 0.08
Control	300	17.20 ± 0.87	24.76 ± 0.57	19.52 ± 0.73	14.74 ± 0.31	2.59 ± 0.01	2.16 ± 0.01
Control	500	19.28 ± 0.58	43.89 ± 0.84	17.02 ± 0.58	26.47 ± 0.57	3.59 ± 0.02	1.73 ± 0.20
ACR52	0	16.96 ± 0.66	18.62 ± 0.32	23.88 ± 0.84	14.28 ± 0.38	1.87 ± 0.06	2.66 ± 0.09
ACR52	300	20.80 ± 0.55	28.33 ± 0.32	18.84 ± 1.10	19.66 ± 0.29	2.62 ± 0.00	1.79 ± 0.11
ACR52	500	18.28 ± 0.58	44.01 ± 0.32	16.61 ± 0.53	23.75 ± 0.31	2.65 ± 0.04	1.62 ± 0.06
EAL318	0	14.32 ± 0.29	31.07 ± 0.55	26.34 ± 0.67	31.01 ± 0.33	2.61 ± 0.03	2.75 ± 0.23
EAL318	300	15.35 ± 0.73	41.53 ± 0.43	19.71 ± 1.07	30.74 ± 0.46	3.99 ± 0.00	2.12 ± 0.04
EAL318	500	26.86 ± 0.71	44.51 ± 0.86	14.94 ± 2.02	30.72 ± 0.39	4.06 ± 0.00	1.48 ± 0.06
EAR195	0	14.11 ± 0.54	31.75 ± 1.45	27.84 ± 1.41	29.08 ± 0.59	1.28 ± 0.33	2.96 ± 0.04
EAR195	300	15.82 ± 0.30	38.24 ± 0.31	23.11 ± 0.60	31.07 ± 0.57	2.86 ± 0.05	2.40 ± 0.03
EAR195	500	17.93 ± 0.53	40.35 ± 0.48	19.68 ± 0.78	32.87 ± 0.97	3.99 ± 0.18	2.09 ± 0.03
C3	0	10.29 ± 0.49	28.55 ± 0.61	34.41 ± 0.19	30.69 ± 0.52	1.12 ± 0.00	2.99 ± 0.04
C3	300	14.25 ± 0.24	35.39 ± 0.37	17.36 ± 0.91	32.70 ± 0.39	1.42 ± 0.10	1.81 ± 0.05
C3	500	19.02 ± 0.57	46.75 ± 0.65	14.98 ± 0.88	38.84 ± 0.48	1.59 ± 0.13	1.37 ± 0.06

Supplementary Table 1. Effects of selected mangrove-derived endophytic bacteria and the three-strain consortium C3 on root antioxidant-enzyme activities and oxidative-damage indicators of peanut under salinity. Peanut cv. JL24 plants were inoculated with ACR52, EAL318, or EAR195, or with the three-strain consortium C3 comprising ACR52, EAL318, and EAR195; uninoculated plants served as the control. Plants were exposed to 0, 300, and 500 mM NaCl. Root biochemical parameters included superoxide dismutase (SOD), catalase (CAT), ascorbate peroxidase (APX), peroxidase (POD), hydrogen peroxide (H_2O_2), and malondialdehyde (MDA). Values represent mean ± SE of three independently treated pots per bacterial treatment × NaCl combination (n = 3), with each pot considered the biological replicate and experimental unit.
